# Effect of non-surgical periodontal therapy on glycemic control of type 2 diabetes mellitus: a systematic review and Bayesian network meta-analysis

**DOI:** 10.1186/s12903-019-0829-y

**Published:** 2019-08-06

**Authors:** Ruoyan Cao, Qiulan Li, Qiqi Wu, Mianfeng Yao, Yu Chen, Hongbo Zhou

**Affiliations:** 1 Department of Prosthodontics, Xiangya Stomatological Hospital & School of Stomatology, Central South University, 72 Xiangya Road, Changsha, 410000 China; 2 Department of Stomatology, The Second Xiangya Hospital, Central South University, 139 Middle Renmin Road, Changsha, 410011 China; 3 Department of Operative Dentistry and Endodontics, Xiangya Stomatological Hospital & School of Stomatology, Central South University, 72 Xiangya Road, Changsha, 410000 China; 4 Department of Oral Medicine, Xiangya Hospital, Central South University, 87 Xiangya Road, Changsha, 410083 China

**Keywords:** Type 2 diabetes mellitus, Periodontitis, Non-surgical periodontal therapy, Scaling and root planing, Adjuvant therapy

## Abstract

**Background:**

Glycemic control is vital in the care of type 2 diabetes mellitus (T2DM) and is significantly associated with the incidence of clinical complications. This Bayesian network analysis was conducted with an aim of evaluating the efficacy of scaling and root planning (SRP) and SRP + adjuvant treatments in improving glycemic control in chronic periodontitis (CP) and T2DM patients, and to guide clinical practice.

**Methods:**

We searched the Pubmed, Embase, Cochrane Library and Web of Science databases up to 4 May 2018 for randomized controlled trials (RCTs). This was at least three months of the duration of study that involved patients with periodontitis and T2DM without other systemic diseases given SRP. Patients in the control group did not receive treatment or SRP combination with adjuvant therapy. Outcomes were given as HbA1c% and levels fasting plasma glucose (FPG). Random-effects meta-analysis and Bayesian network meta-analysis were conducted to pool RCT data. Cochrane’s risk of bias tool was used to assess the risk of bias.

**Results:**

Fourteen RCTs were included. Most were unclear or with high risk of bias. Compared to patients who did not receive treatment, patients who received periodontal treatments showed improved HbA1c% level, including SRP (the mean difference (MD) -0.399 95% CrI 0.088 to 0.79), SRP + antibiotic (MD 0.62, 95% CrI 0.18 to 1.11), SRP + photodynamic therapy (aPDT) + doxycycline (Doxy) (MD 1.082 95% CrI 0.13 to 2.077) and SRP + laser (MD 0.66 95% CrI 0.1037, 1.33). Among the different treatments, SRP + aPDT + Doxy ranked best. Regarding fasting plasma glucose (FPG), SRP did not show advantage over no treatment (MD 4.91 95% CI − 1.95 to 11.78) and SRP with adjuvant treatments were not better than SRP alone (MD -0.28 95% CI -8.66, 8.11).

**Conclusion:**

The results of this meta-analysis seem to support that periodontal treatment with aPDT + Doxy possesses the best efficacy in lowering HbA1c% of non-smoking CP without severe T2DM complications. However, longer-term well-executed, multi-center trails are required to corroborate the results.

**Electronic supplementary material:**

The online version of this article (10.1186/s12903-019-0829-y) contains supplementary material, which is available to authorized users.

## Background

Periodontitis is a chronic inflammatory disease caused by pathogens in the surrounding periodontal tissues, that results in the periodontal pocket formation, clinical attachment loss and alveolar bone resorption and ultimately leads to tooth loss [1, 2]. Epidemiological evidence has shown that periodontitis affects over 50% of the adult worldwide, indicating a dose-response relationship with oral health relates to the quality of life [3, 4]. It is well-known that periodontitis is highly associated with T2DM, and now periodontitis is regarded as the sixth complicated form of T2DM [5]. Compared to non-diabetics, patients with diabetes present worse clinical manifestation of periodontitis [6]. Besides, moderate to severe periodontitis increases the risk of T2DM and leads to poor glycemic control in diabetics [7, 8].

Glycemic control is vital in the care of T2DM and is significantly associated with the incidence of clinical complications. One percent reduction in HbA1c% is associated with 14% reductions in risk of myocardial infarction, 21% for deaths related to diabetes and 37% for microvascular complications [9]. Studies indicate that scaling and root planing (SRP) or SRP plus adjuvant treatment could improve glycemic control in patients with T2DM and CP, but no effective conclusion has been reached regarding the best treatment. Besides, there is conflicting evidence regarding glycemic control of various adjuvant treatments in SRP. For instance, SRP followed by locally delivered Atorvastatin (ATV) did not show a reduction of HbA1c% compared to SRP [10], while additional benefits were found after adjuvant therapy with aPDT [11]. Previous meta-analyses usually compared periodontal treatment with no treatment [12]. Few of meta-analyses have focused on adjuvant therapy, but adjuvant therapy is limited to a single type, such as aPDT [13] and systemic antibiotics [14, 15]. Therefore, a more comprehensive study is needed to clarify whether SRP or SRP with adjuvant treatments could improve glycemic control in patients diagnosed with T2DM, and potentially find the best treatment to provide evidence for clinical practice.

This Bayesian network analysis aimed to address the following focused question based on the Population, Intervention, Comparison, Outcomes, Study Design (PICOS) schema: “In chronic periodontitis with T2DM, does periodontal treatment with/without adjuvant treatment compared to no treatment or periodontal treatment with adjuvant treatment compared to periodontal treatment alone, result in better glycemic control in randomized controlled clinical trials?”

## Methods

This review was not registered as a priori protocol but followed the PRISMA and the PRISMA extension for Systematic reviews and Meta-Analyses network meta-analysis, respectively.

### Eligibility criteria

The studies were considered eligible for inclusion if they met the following criteria based on PICO schema:

- Participants: Adult patients (aged ≥30 years) with no gender, age and career predilection diagnosed as periodontitis and T2DM
- Intervention: Comparing SRP with no treatment, or comparing SRP with SRP plus adjuvant treatment, or comparing SRP plus adjuvant therapy with different adjuvant therapies
- Outcoming: HbA1c% and fasting plasma glucose (FPG) (mean change in parameters from baseline to follow-up visits)
- Study design: Randomized clinical trial (RCT)
- Follow up: At least three months
- Language: English

### Exclusion criteria

- Split-mouth randomized controlled clinical trial
- Pregnancy and lactation
- Current smoking and smoking within the past 5 years
- Studies including subjects who had systemic conditions (except T2DM) and major complications of T2DM
- Periodontal treatment and antibiotic use within the previous three months
- Periodontal support therapy within three months
- Sample sizes of each group less than 10
- Studies that did not report the value of HbA1c and FPG

### Information sources and literature search

We searched the Pubmed, Embase, Cochrane Library and Web of Science Databases from inception to 4 May 2018. The following MeSH terms/free terms and their combinations were searched to find potentially eligible studies (Additional file 1). Also, the reference lists of relevant articles and relevant systematic reviews were manually searched to find other potentially eligible studies.

### Study selection

Two independent reviewers (R.Y. Cao and Q.L. Li) screen identified eligible studies based on their titles/abstracts and full texts. Any disagreements were resolved by discussion or through an adjudication by a third review (M.F. Yao).

### Data extraction and data items

Two independent investigators (R.Y. Cao and Q.L. Li) extracted and recorded relevant data from eligible studies by pre-designed data-extraction forms: study characteristics (first author, publication year, country), patient characteristics (age, sex,inclusion criteria of T2DM and periodontitis, T2DM duration, T2DM treatment), intervention and control treatment protocols, sample size, outcome details (detection method of HbA1c%, ΔHbA1c%, ΔFPG), follow-up period, adverse events. Inconsistencies were settled through discussions until an agreement was reached.

### Risk of bias in individual trails

The risk of bias of included studies was assessed independently by two reviewers (R.Y. Cao and Q.L. Li) with Cochrane Collaboration tool. The items for Cochrane tool included random sequence generation; allocation concealment; blinding of participants and personnel assessment; incomplete outcome data; selective outcome reporting and other bias. We did not assess the blinding of the outcome because our outcomes (HbA1c% and FPG) were objective indicators. Inconsistencies were settled through discussions until an agreement was reached.

### Outcomes and data synthesis

The treatment outcomes were the absolute difference (AD) in HbA1c% and FPG at three to four months after periodontal treatment. When the standard deviations (SD) for the outcomes were not available, it was calculated by assuming that the correlation coefficient was 0.5 as previously described [16, 17].

The data used was a follow-up of 3–4 months. First, a random-effects pairwise meta-analysis was performed with the Stata 14.2 (Stata Corporation, College Station, TX, USA). The mean difference (MD) and 95% confidence intervals (CIs) were used to compare continuous variables as previously described [12]. Second, we did a Bayesian network analysis by using Markov chain Monte Carlo methods via GeMTC package 0.8 implemented in R 3.2.2. We also assessed study design information and patient characteristics to evaluate the transitivity assumption for reliable data pooling with sufficient similarity between the included trials [18, 19]. Both non-informative uniform and normal prior distributions were used throughout the network meta-analysis [20]. Markov chain Monte Carlo methods with four chains of 300,000 iterations after a burn-in phase of 180,000 iterations were used to gain MD and 95% credible intervals (CrIs). We used CrIs beyond the null value to assess significance. We also performed a sensitivity analysis by setting a uniform prior standard deviation (“std.dev”, “dunif”, 0, 2). Model selection (fixed vs random effects) was based on the assessment of the deviance information criterion (DIC) [21]. We then reported the results of the random-effects model. Brooks-Gelman-Rubin diagnostic plot was used to assess model convergence of network analysis [19]. We also ranked the different treatments in terms of lowering the HbA1c% level using the same methods [20].

The goodness of fit of the model assessed the consistency assumption of this network analysis and it was tested by calculating the posterior mean residual deviance (Dbar). When the Dbar was similar to the number of data points in the study, the model was taken into account for fitting the data well [22]. Moreover, we also examined the pooled effects from traditional pairwise meta-analysis and network meta-analysis to further verify the consistency of the network. Inconsistency was assessed by comparing direct evidence with indirect evidence from the entire network on each node (node-splitting analysis) with *p* < 0.05 [23]. Heterogeneity was assessed by I^2^ calculation. To verify the robustness of our analyses, we performed a sensitivity analysis by excluding studies with HbA1c% > 9 or < 7 as the baseline. We also conducted comparison-adjusted funnel plots to assess publication bias. All statistical analyses were performed using Review Manager 5.3 (Cochrane Collaboration, Oxford, UK), R 3.2.2 (R Foundation for Statistical Computing, Vienna, Austria) and Stata 14.2 (Stata Corporation, College Station, TX, USA).

### Quality of network meta-evidence

We assessed the quality of evidence for direct estimates based on the GRADE criteria [24] using five items (risk of bias, imprecision, indirectness, inconsistency and publication bias). We used an approach that proposed by Puhan et al. and Brignardello-Petersen et al. to assess the quality of evidence for indirect and network estimates.

## Results

### Study selection

**Fig. 1:**
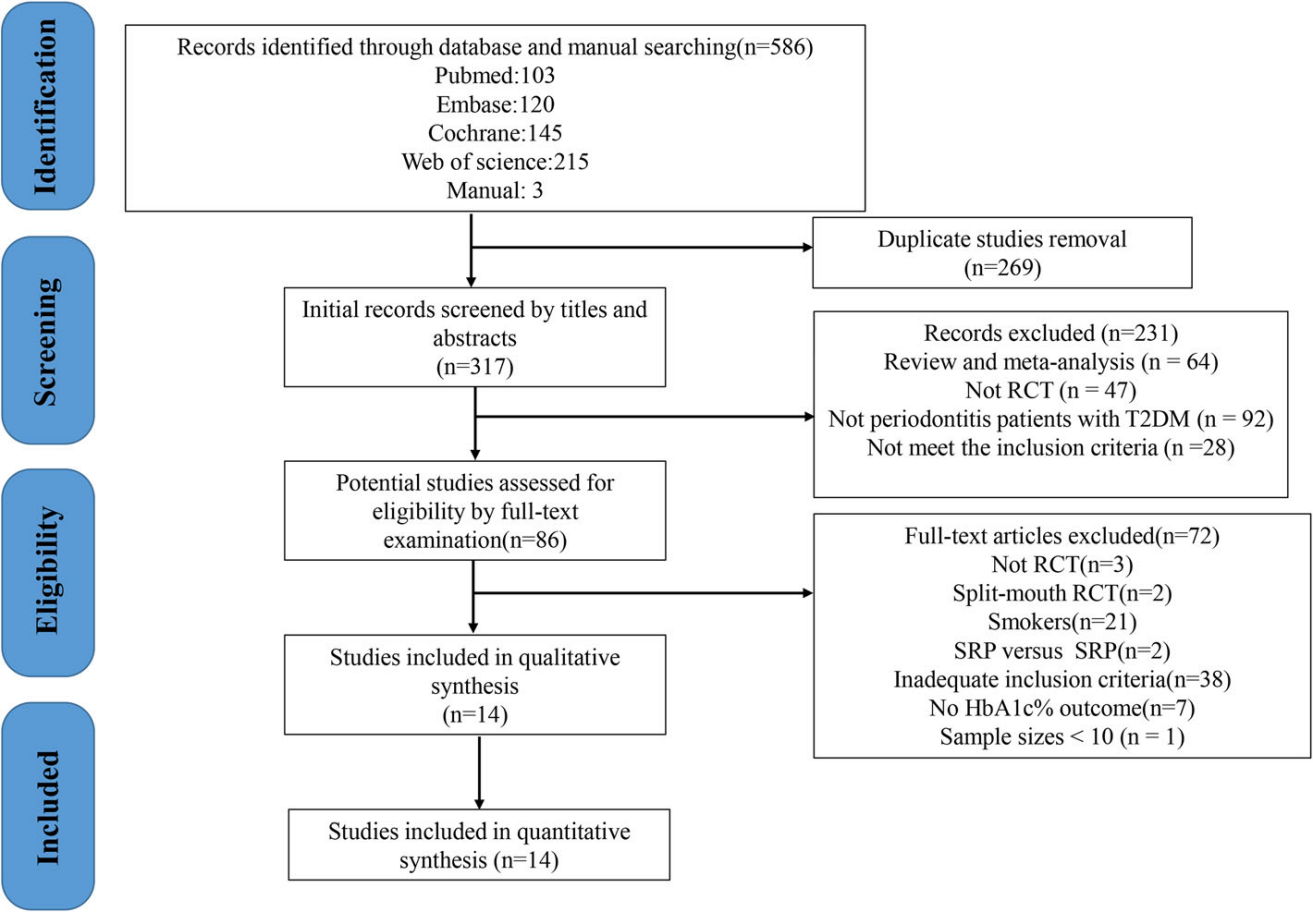
Flow chart of articles search and screening process

The literature search identified 586 possibly eligible articles, 86 articles were fully accessed after excluding duplicates and unsuitable studies by title/abstract, while 72 of them were not considered eligible for inclusion (Additional file 2). A total of 14 trials with 629 patients were included in this network meta-analysis. Figure 1 shows phases of the screening process.

### Characteristics of included studies

**Fig. 2:**
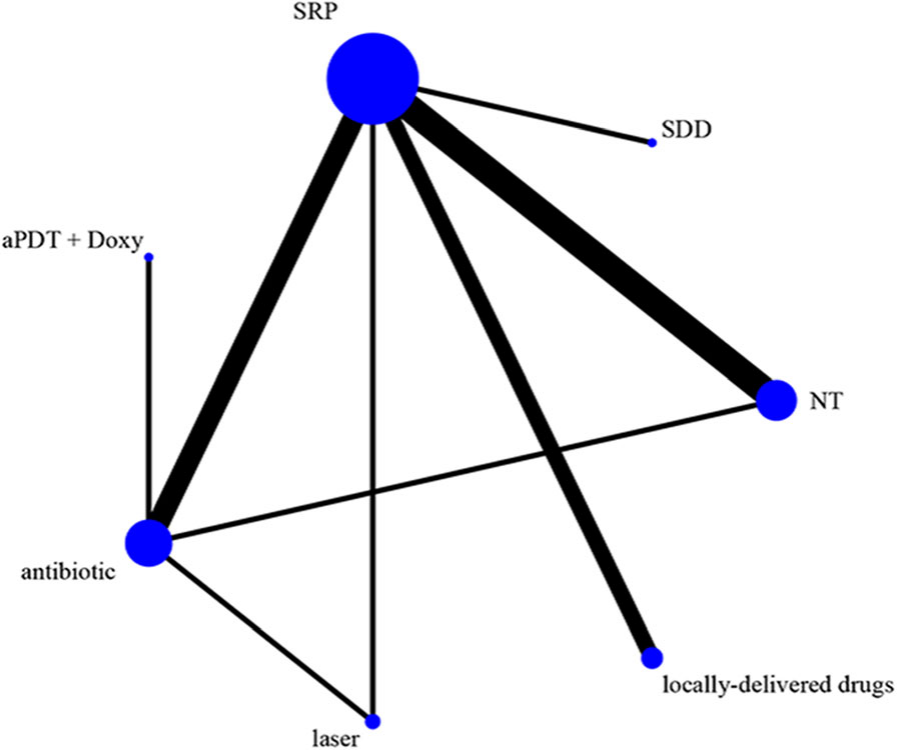
Network of the interventional comparisons for the Bayesian network analysis. The size of the nodes is proportional to the number of subjects (sample size) randomized to receive the therapy. The width of the lines is proportional to the number of trials comparing each pair of treatments. aPDT, antimicrobial photodynamic therapy; Doxy, doxycycline; antibiotics (Doxy, metronidazole + amoxicillin); local, locally-delivered drugs (atorvastatin gel, chlorhexidine gel, simvastatin gel); laser (diode laser, aPDT); SDD, subantimicrobial dose doxycycline; SRP, scaling and root planing, NT, no treatment

Tables 1 and 2 presented the main characteristics of the 14 included studies. All studies were RCTs, and were followed up for 3–12 months. All the patients had T2DM and chronic periodontitis, but the diagnostic criteria of T2DM were not reported in most studies. The T2DM duration was from 4 to 11.8 years. T2DM treatments mainly included diet and insulin supplementation or oral hypoglycemic agents. The baseline of HbA1c% varied widely, from 6.2 to 10.4. Periodontal treatments were performed by SRP, within 24 h - 2 weeks. Most studies gave oral hygiene instruction to subjects. All studies reported the outcomes of HbA1c%, few reported on FPG. Four studies did not report adverse events, and three studies [30, 32, 33] showed side effects, such as taste perception, dry mouth, headache, diarrhoea. Ten studies reported the method of HbA1c% determination. The network meta-analysis included 7 treatments (Fig. 2), including SRP (*n* = 280), no treatment (*n* = 76), SRP + antibiotic (doxycycline (Doxy), metronidazole + amoxicillin, Doxy) (*n* = 130), subantimicrobial dose doxycycline (SDD) (*n* = 17), locally-delivered drugs (atorvastatin gel, simvastatin gel, chlorhexidine gel) (*n* = 66), laser (diode laser, aPDT) (*n* = 45) and SRP + Doxy + aPDT (*n* = 15). The plots indicated that most of the direct evidence between different adjuvantive treatments was lacking.

**Table 1:** Characteristics of studies included in the network meta-analysis

First author, year	Country	Inclusion criteria of T2DM	T2DM duration (year)	T2DM treatment	Age (years)	Female/male	Intervention	Control
Singh (2008) [25]	India	T2DM	na	na	> 30	na	A: SRP + Doxy (100 mg bid for day 1 and then 100 mg/day for 13 days) (n = 15) B: SRP (n = 15)	No treatment (n = 15)
Gilowski (2012) [26]	Poland	T2DM diagnosed for > 6 months	T: 4.0 (1.5–13.0) C: 5.0 (3.0–18.0)	diet regimen, insulin, and or oral hypoglycemic drugs	T: 57.6 ± 8.0 C: 56.0 ± 9.0	T: 10/ 7 C: 8/ 9	SRP + SDD (20 mg bid for 3 months) (n = 17)	SRP + placebo (n = 17)
Moeintaghavi (2012) [27]	Iran	Diagnosis of T2DM with glycated HbA1c values over 7%	na	oral hypoglycemic drugs (no insulin)	Female: 48.1 ± 3 Male: 52.48 ± 3	T: 13/ 9 C: 7/ 11	SRP (n = 22)	No treatment (n = 18)
Gaikwad (2013) [28]	India	T2DM	na	Received antidiabetic therapy	na	16/ 34	SRP + Doxy (100 mg/d for15 days) (n = 25)	SRP (n = 25)
Pradeep (2013) [29]	India	Well-controlled type 2 DM (American Diabetic Association in 2011 and glycated hemoglobin levels)	na	na	30 - 50	18/ 20	SRP + SMV gel (1.2%) (n = 17)	SRP + placebo (n = 18)
Santos (2013) [30]	Brazil	T2DM diagnosed > 5 years	T: 6.3 ± 0.8 C: 6.8 ± 1.1	Diet: 1/ 0 Diet + insulin: 1/ 4 Diet + oral hypoglycemic agents: 14/ 14 Diet + oral hypoglycemic agents + insulin: 3/ 1	T: 50.3 ± 9.5 C: 53.9 ± 10.8	T: 15/ 4 C: 13/ 6	SRP + CHX gel (1%) + CHX solution (0.12%) rinse (bid/day for 60 days) (n = 19)	SRP + placebo gel + placebo solutions rinse (n = 18)
Telgi (2013) [31]	India	T2DM	na	oral hypoglycemic agents	35 - 45	na	A: SRP + 0.12% CHX mouthwash (once daily) and brush (twice daily) (n = 20) B: 0.12% CHX mouthwash (once daily) and brush (twice daily) (n = 20)	brush (twice daily) (n = 20)
Macedo (2014) [11]	Brazil	T2DM diagnosed for >5 years and HbA1c >7 %	> 5	na	T: 49.4 ± 6.8 C: 48.1 ± 9	T: 9/ 6 C: 10/ 5	SRP + aPDT (10 mg/ml PC) + Doxy (100 mg bid for day 1 and then 100 mg/day for 13 days) (n = 15)	SRP + Doxy (100 mg bid for day 1 and then 100 mg/day for 13 days) (n = 15)
Miranda (2014) [32]	Brazil	T2DM diagnosed for ≥ 5 years, HbA1c ≥ 6.5% ≤ 11%	T: 8.0 ± 3.2 C: 7.4 ± 3.6	Diet and insulin supplementation or oral hypoglycemic agents	T: 54.0 ± 8.2 C: 53.7 ± 8.0	T: 17/ 12 C: 9/ 18	SRP + MTZ + AMX (400/500 mg tid for 14 days) (n = 29)	SRP + placebos (n = 27)
Tsalikis (2014) [33]	Greece	T2DM diagnosed for ≥ 1 year	T: 11.8 ± 5.9 C: 10.2 ± 5.7	na	T: 62.9 ± 10 C: 57.94 ±8.22	T: 13/ 18 C: 15/ 20	SRP+ Doxy (100 mg bid for day 1 and then 100 mg/day for 20 days) (n = 31)	SRP + placebos (n = 35)
Wu (2015) [34]	China	T2DM diagnosed for > 1 year with no medication changes in the last 3 months	T: 4.00 ± 1.76 C: 4.22 ± 1.57	na	T: 54.09 ±6.57 C: 55.52 ±5.22	T: 12/11 C: 10/13	SRP + OHI (n = 23)	OHI (n = 23)
Koçak (2016) [35]	Turkey	TDM2 (5.7 % ≤ glycated hemoglobin (HbA1c) ≤8.5 %) with no alteration in the diabetes treatment in the last year prior to the study	Na	na	T: 51.7 ± 5.2 C: 53.1 ± 5.1	T: 15/15 C: 15/15	SRP + DL (n = 30)	SRP (n = 30)
Kumari (2016) [10]	India	Well-controlled T2DM (American Diabetic Association in 2012 and glycated hemoglobin Levels)	Na	na	40-50	37/38	SRP + ATV gel (1.2%) (n = 30)	SRP + placebo gel (n = 30)
Ramos (2016) [36]	Brazil	Type 2 DM diagnosed for > 5 years and HbA1c >7%	>5	na	T: 48.9 ± 9.5 C: 49.3 ± 7.4	T: 8/7 C: 8/7	SRP + aPDT (10 mg/ml PC) (n = 15)	SRP + Doxy (100 mg bid for day 1 and then 100 mg/day for 13 days) (n = 15)

*SRP* scaling and root planing, *Doxy* doxycycline, *SDD* subantimicrobial dose doxycycline, *SMV* simvastatin, *CHX* chlorhexidine, *aPDT* antimicrobial photodynamic therapy, *AMX* amoxicillin, *MTZ* metronidazole, *ATV* atorvastatin, *DL* diode laser, *PC* phenothiazine chloride solution, *na* not available

**Table 2:** Outcomes of studies included in the network meta-analysis

First author, year	Inclusion criteria of chronic periodontitis	Method of HbA1c% determination	Initial HbA1c (%) (T vs. C)	Outcome (ΔHbA1c%) (T vs. C)	Outcome (ΔFPG) (T vs. C)	Follow up (m)	Adverse events (T vs. C)
Singh (2008) [25]	Moderate to advanced periodontitis; at least 30% teeth with PPD ≥ 4 mm	Liquid chromatography method	TA: 8.3 ± 0.7 TB: 7.9 ± 0.7 C: 8.08 ± 0.7	TA: 0.8 ± 0.66 TB: 0.6 ± 0.66 C: -0.02 ± 0.72	TA: 4.16 ± 11.55 TB: 4 ± 14.45	3	No
Gilowski (2012) [26]	Periodontitis; at least four nonadjacent sites with PPD ≥ 4 mm	Turbidimetric inhibition immunoassay	T: 6.7 (6.3–7.0) C: 6.2 (6.0–7.8)	T: 0 ± 0.9 C: 0.1 ± 1.41		3	No
Moeintaghavi (2012) [27]	Mild to moderate periodontitis (American Academy of Periodontology)	Cobas Integra 700 apparatus (Roche Diagnostics, Germany)	T: 8.15 ± 1.18 C: 8.72 ± 2.22	T: 0.74 ± 1.18 C: -0.25 ± 2.05	T: 17.5 ± 48.91 C: -9.78 ± 38.02	3	na
Gaikwad (2013) [28]	Chronic generalized periodontitis	na	T: 8.38 ± 0.89 C: 8.06 ± 1.10	T: 1.38 ± 0.83 C: 0.95 ± 1.05	na	4	No
Pradeep (2013) [29]	Chronic periodontitis; PPD ≥ 5 mm or CAL ≥ 4 mm and vertical bone loss ≥ 3mm on intraoral periapical radiographs	na	T: 6.66 ± 0.11 C: 6.71 ± 0.13	3m: T: 0.02 ± 0.12 C: 0.03 ± 0.13 6m: T: 0.01 ± 0.12 C:0.03 ± 0.13 9m: T: 0.03 ± 0.11 C: 0.05 ± 0.13	na	9	No
Santos (2013) [30]	Generalized chronic periodontitis (Armitage 1999); at least 30% of sites with PPD and CAL ≥ 4 mm	high-performance liquid chromatography	T: 10 ± 2.41 C: 10.4 ± 2.9	3m: T: 0.78 ± 3.5 C: 0.68 ± 2.5 6m: T: 0.19 ± 2.9 C: 0.74 ± 4.1 12m: T: 0.35 ± 3.4 C: 1.4 ± 3.0	3m: T: 5.4 ± 65.09 C: 1.4 ± 77.31 6m: T: 5.5 ± 57.34 C: 15.4 ± 80.06 12m: T: 7.2 ± 72.37 C: 20.6 ± 79.15	12	T: 7, C: 12 reported taste perception change/ dry mouth/ staining
Telgi (2013) [31]	mild to moderate periodontitis; PD: 4 – 5 mm	na	TA: 7.68 ± 0.63 TB: 7.56 ± 0.59 C: 7.74 ± 0.59	TA: 0.58 ± 0.27 TB: 0.25 ± 0.14 C: 0.004 ± 0.12	TA: 2.88 ± 1.07 TB: 1.29 ± 0.53 C: 0.42 ± 0.71	3	na
Macedo (2014) [11]	Chronic periodontitis, at least one site with PPD ≥ 5 mm on each quadrant, two teeth with CAL ≥ 6 mm	automated immunoturbidimetric method	T: 8.6 ± 1.1 C: 8.0 ± 0.93	T: 0.87 ± 0.9 C: 0.41 ± 0.84	na	3	No
Miranda (2014) [32]	the sites with PPD and CAL ≥ 4 mm, a minimum of six teeth with at least one site with PPD and CAL ≥ 5 mm, BOP	high-performance liquid chromatography	T: 8.53 ± 1.56 C: 8.99 ± 1.63	3m: T: -0.07 ± 1.83 C: 0.05 ± 1.67 6m: T: 0.04 ± 1.94 C: -0.08 ± 1.66 12m: T: -0.24 ± 2.54 C: 0.59 ± 1.81	3m: T: 5.46 ±38.30 C: 8.71 ± 40.41 6m: T: 6.19 ±34.01 C: 5.95 ± 45.50 12m:T:1.59± 36.53 C: 6.9 ± 47.09	12	Diarrhea: T: 7, C:3 Headache: T: 4, C: 1 Metallic taste: T: 4, C: 2 Nausea/Vomiting: T: 5, C: 2
Tsalikis (2014) [33]	Moderate or advanced periodontitis; six pockets > 5mm, CAL > 3mm with radiographic bone loss	A1CNow + Multitest HbA1c system	T: 6.70 ± 0.61 C: 6.89 ± 0.60	3m: T: 0.08 ± 0.58 C: -0.07 ± 0.88 6m: T: 0.22 ± 0.67 C: 0.09 ± 0.77	na	6	C: 1 reported Dizziness and difficulty to swallow
Wu (2015) [34]	mean CAL > 1mm (American Academy of Periodontology criteria)	immunoturbidimetry	T: 7.41 ± 0.20 C: 7.39 ± 0.16	3m: T: 0.01 ± 0.19 C: 0.01 ± 0.16 6m: T: 0.32 ± 0.17 C: 0.03 ± 0.17	na	6	na
Koçak (2016) [35]	≥ 8 sites with PPD ≥5mm	na	T: 6.9 ± 0.7 C: 6.5 ± 0.6	T: 0.41 ± 0.19 C: 0.22 ± 0.25	na	3	na
Kumari (2016) [10]	Chronic periodontitis; PPD ≥ 5 mm or CAL ≥ 4 mm, and vertical bone loss ≥ 3 mm on intraoral periapical radiographs	na	T: 6.72 ± 0.13 C: 6.75 ± 0.12	3m: T: 0.04 ± 0.14 C: 0.03 ± 0.13 6m: T: 0.06 ± 0.13 C: 0.04 ± 0.13 9m: T: 0.07 ± 0.13 C: 0.06 ± 0.13	na	9	No
Ramos (2016) [36]	At least one site with PPD ≥ 5 mm on each quadrant and two teeth with CAL ≥ 6 mm	High pressure liquid chromatography	T: 10.6 ± 1.99 C: 9.69 ± 1.68	T: 0.99 ± 1.00 C: 0.76 ± 0.73	na	3	No

*CAL* clinical attachment loss, *PPD* probing pocket depth, *BOP* bleeding on probe, *na* not avail

### Risk of bias in included studies

**Fig. 3:**
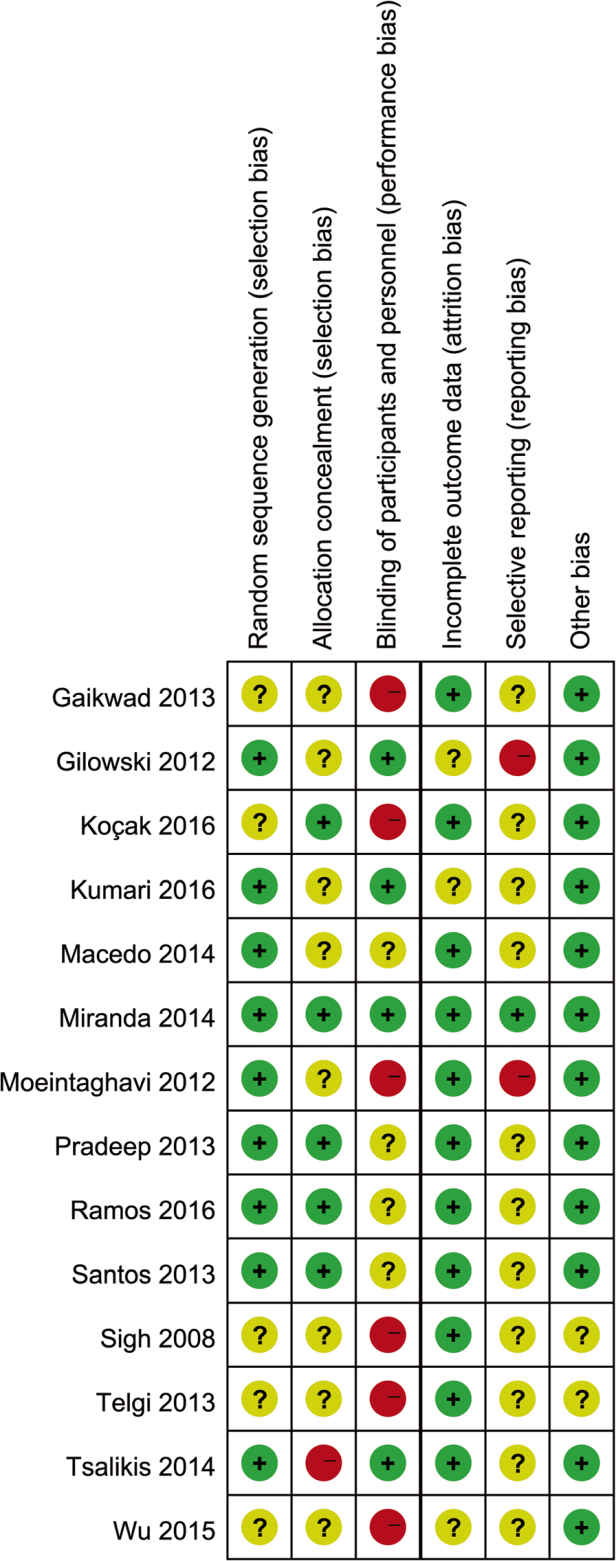
Judgements about each risk of bias item for each included study

Figure 3 presented reviewers’ judgments on the risk of bias in the referred RCTs. Most included studies were found to have methodological issues, with the most problematic domains being the allocation concealment (unclear or high risk in 35.7% of studies). Though the included studies were RCTs, 35.7% studies did not report the details of the randomized method. One study had an low risk of selective outcome reporting bias. In addition, two studies also might have had other bias, because we could not judge if there were baseline imbalances between groups (Additional file 3).

### Efficacy of different treatments for the reduction of HbA1c% in subjects with CP and T2DM

In traditional meta-analysis (Table 3), SRP + laser showed additional benefits compared to SRP alone (0.19 [0.08, 0.30]). There was statistically significant reduction in HbA1c% after treated with SRP + antibiotic (0.82 [0.33, 1.31]) compared to no treatment. In addition, there was no other significant difference was observed among the rest of the comparisons. The overall heterogeneity was high (Global I^2^ = 83.2), but mainly between SRP and no treatment (I^2^ = 94.2), while the heterogeneity was lower in other subgroups (I^2^ = 0).

**Table 3:** Comparison of outcomes between traditional meta-analysis and Bayesian network meta-analysis

treatment comparison	traditional meta-analysis	network meta-analysis
SRP + Antibiotic vs. SRP	0.21 (-0.03, 0.45)	0.20 (-0.17, 0.56)
SRP + Laser vs. SRP	0.19 (0.08, 0.30)	0.25 (-0.24, 0.78)
SRP + locally-delivered drug vs. SRP	0.00 (-0.05, 0.05)	0.0026 (-0.41, 0.43)
SRP + SDD vs. SRP	-0.10 (-0.90, 0.70)	-0.10 (-1.1, 0.89)
SRP + aPDT + Doxy vs. SRP + Antibiotic	0.46 (-0.16, 1.08)	0.46 (-0.40, 1.31)
SRP + Laser vs. SRP + Antibiotic	0.23 (-0.40, 0.86)	0.053 (-0.48, 0.61)
SRP vs. No treatment	0.45 (0.00, 0.89)	0.40 (0.088, 0.80)
SRP + Antibiotic vs. No treatment	0.82 (0.33, 1.31)	0.61 (0.16, 1.11)

*SRP* scaling and root planing, *SDD* subantimicrobial dose doxycycline, *aPDT* antimicrobial photodynamic therapy, *Doxy* doxycycline

**Fig. 4:**
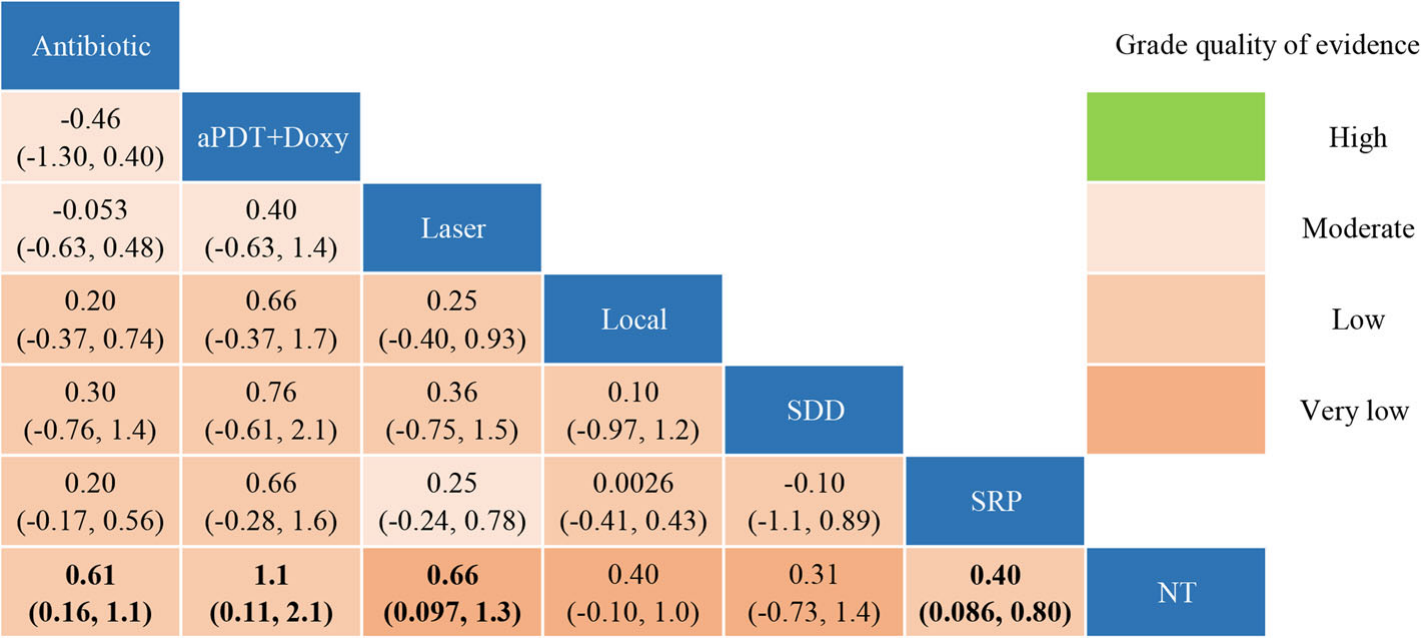
Multiple-treatment comparisons and the quality of evidence for ΔHbAlc%. aPDT, antimicrobial photodynamic therapy; Doxy, doxycycline; antibiotics (Doxy, metronidazole + amoxicillin); local, locally-delivered drugs (atorvastatin gel, chlorhexidine gel, simvastatin gel); laser (diode laser, aPDT); SDD, subantimicrobial dose doxycycline; SRP, scaling and root planing, NT, no treatment

**Fig. 5:**
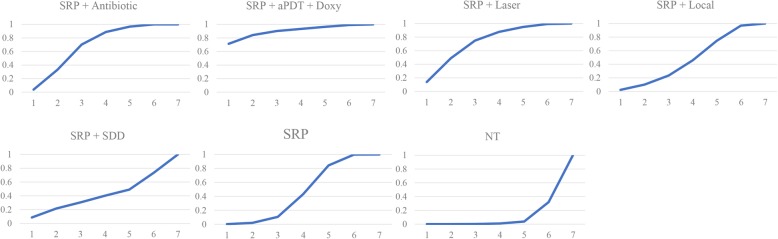
The rank of different treatments. aPDT, antimicrobial photodynamic therapy; Doxy, doxycycline; antibiotics (Doxy, metronidazole + amoxicillin); local, locally-delivered drugs (atorvastatin gel, chlorhexidine gel, simvastatin gel); laser (diode laser, aPDT); SDD, subantimicrobial dose doxycycline; SRP, scaling and root planing, NT, no treatment

In network meta-analysis, sensitivity analysis indicated that both non-informative uniform and normal prior distributions were properly used (Additional file 4). SRP + antibiotic (0.61 [0.16, 1.1]), SRP + aPDT + Doxy (1.1 [0.11, 2.1]), SRP + laser (0.66 [0.097, 1.3]) and SRP alone (0.40 [0.086, 0.80]) was significantly better than no treatment in improving HbA1c%, separately (Fig. 4). No other significant difference was observed among the rest of the comparisons. The probabilities the most effective treatment methods of decreasing HbA1c% was SRP + aPDT + Doxy (71.2%), followed by SRP + laser (13.6%), SRP + SDD (8.6%), SRP + antibiotic (3.8%), SRP (0.2%) and no treatment (0.02%) (Fig. 5). The Global I^2^ was 86.18, except that between SRP and no treatment or SRP + laser were 95.63 and 23.56, respectively, the other subgroups were 0 (Table 4).

**Table 4:** Analysis of heterogeneity

t1	t2	i2.pair	i2.cons	incons.p
HbA1c%				
Per-comparison I-squared				
SRP + Antibiotic	SRP + aPDT + Doxy	NA	NA	NA
SRP + Antibiotic	SRP + Laser	NA	0.00	0.62
SRP + Antibiotic	NT	NA	0.00	0.587
SRP + Antibiotic	SRP	0.00	0.00	0.98
SRP + SDD	SRP	NA	NA	NA
SRP + Laser	SRP	NA	23.56	0.77
SRP + Locally-delivered drugs	SRP	0.00	0.00	NA
NT	SRP	95.43	95.63	NA
Global I-squared		88.11	86.18	

*SRP* scaling and root planing, *SDD* subantimicrobial dose doxycycline, *t1* treatment 1, *t2* treatment 2, *i2.pair* i-square of pair-wise meta-analysis, *i2.cons* i-square of network meta-analysis, *incons.p* inconsistency p-values for pairwise and network meta-analysis, *NA* not applicable

### Efficacy of different treatments for the reduction of FPG in subjects with CP and T2DM

We only did a pairwise meta-analysis, because there were only 5 available studies. SRP was not better than no treatment in FPG reduction (4.91 [− 1.95, 11.78], I^2^ = 46.6%) (Additional file 5). Moreover, no significant difference was observed between SRP + adjuvant and SRP (− 0.28 [− 8.66, 8.11], I^2^ = 0.0%).

### Model fit and evaluation of consistency

The model fitted the data well for the Dbar approximated data points in both outcomes (Additional file 6), and the effects of most comparisons between pairwise and network meta-analysis were similar in the relevant CI or CrI (Table 3). In addition, the node-splitting analysis also showed that there was no inconsistency among direct, indirect and network outcomes with *P* > 0.05 (Table 5).

**Table 5:** The results of node-spitting analysis

comparison	*p*-value	MD (95%CrI)
HbA1c%		
SRP + Antibiotic vs. SRP + Laser		
direct	0.61455	0.23 (-0.66, 1.1)
indirect		-0.059 (-0.83, 0.72)
network		0.043 (-0.49, 0.62)
SRP + Antibiotic vs. No treatment		
direct	0.5605667	-0.82 (-1.7, 0.036)
indirect		-0.53 (-1.2, 0.090)
network		-0.62 (-1.1, -0.18)
SRP + Antibiotic vs. SRP		
direct	0.6489667	-0.22 (-0.65, 0.22)
indirect		0.040 (-1.1, 1.2)
network		-0.22 (-0.58, 0.15)
SRP + Laser vs. SRP		
direct	0.5950500	-0.19 (-0.85, 0.47)
indirect		-0.48 (-1.5, 0.50)
network		-0.26 (-0.79, 0.23)

*SRP* scaling and root planing

### Sensitivity analysis and publication bias

**Fig. 6:**
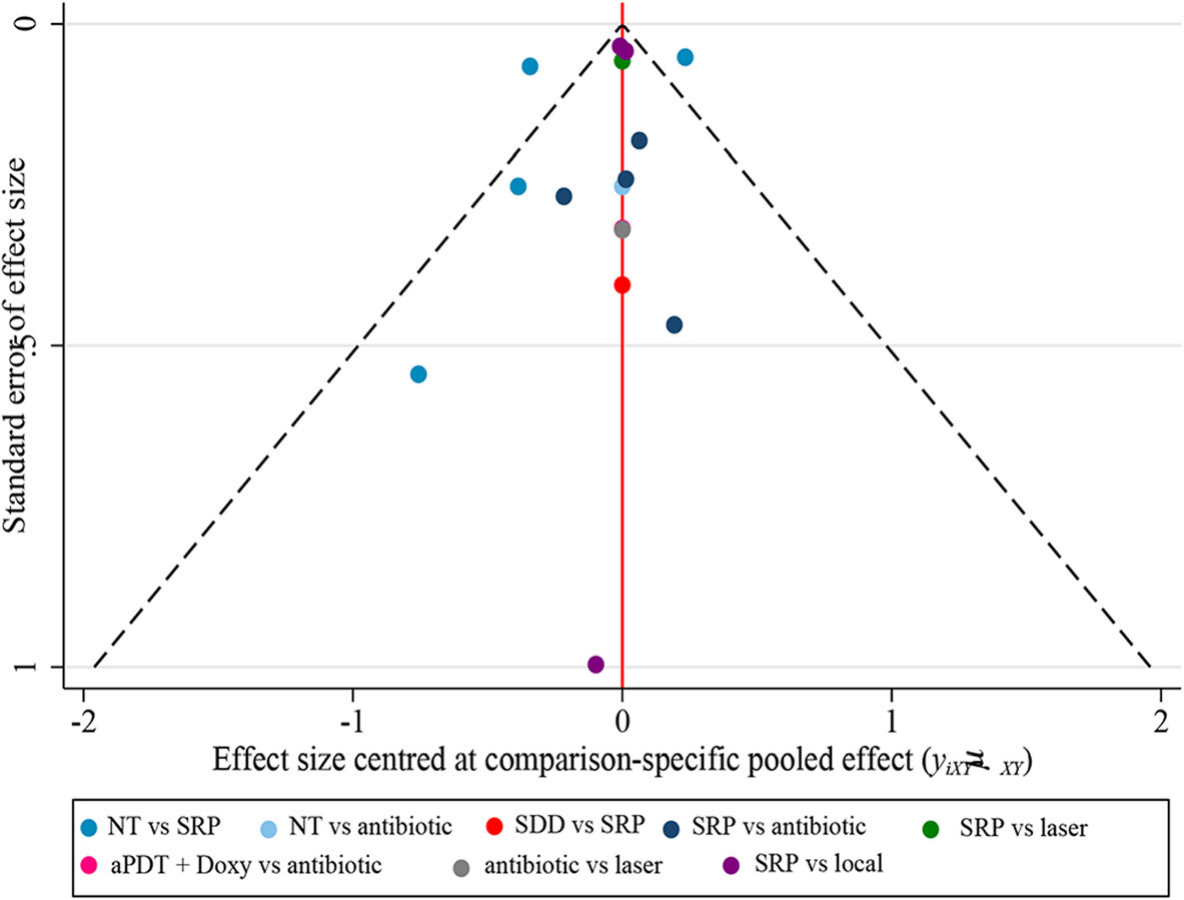
Publication bias assessment for ΔHbAlc%. aPDT, antimicrobial photodynamic therapy; Doxy, doxycycline; antibiotics (Doxy, metronidazole + amoxicillin); local, locally-delivered drugs (atorvastatin gel, chlorhexidine gel, simvastatin gel); laser (diode laser, aPDT); SDD, subantimicrobial dose doxycycline; SRP, scaling and root planing, NT, no treatment

To explore whether the baseline level affects the final effects, we excluded studies with relative higher HbA1c% (> 9%) or relative lower HbA1c% (< 7%). The results were shown in Additional file 7. When we excluded the studies with HbA1c% > 9%, no major changes were found except for SRP + laser versus no treatment, from 0.66 [0.097, 1.3] to 0.59 [− 0.15, 1.4]. Nevertheless, if we excluded studies with HbA1c% < 7%, no significant difference was found any treatment comparisons. The result of the comparison-adjusted funnel plot of HbA1c% reduction revealed there may be publication bias owing asymmetry (Fig. 6).

### Quality of network meta-evidence

We used Grade approach to assess the quality of evidence and the results were presented in Fig. 4. Most of the comparisons were found to be of low quality evidence, four comparisons (SRP + antibiotic vs. SRP + aPDT + Doxy, SRP + laser vs. SRP/SRP + antibiotic/aPDT + Doxy) were considered to have moderate quality of evidence and three comparisons (SRP + laser/ local-delivery drugs) were considered as low-quality of evidence. These indicate that we have limited confidence in these recommendations, and future research may change them.

## Discussion

This network meta-analysis of 14 RCTs including 629 patients that compared the HbA1c% reduction of different treatments indicated that all the treatments might improve HcA1c% than no treatment except for SDD or local-delivery drugs adjunctive to SRP. Furthermore, among the treatments, SRP + aPDT + Doxy was the most effective. No evidence of effectiveness was found in the lowering of FPG.

Evidence-based data of the periodontal treatment effect on glycemic control in T2DM are limited. Although some studies have compared SRP with no treatment or SRP + adjuvant treatment, there was still a lack of sufficient data comparing the different adjuvant treatments. Our study included all periodontal treatments that met our inclusion criteria and made a more overall evaluation. The results from network meta-analysis and traditional meta-analysis were largely consistent. Even so, laser might improve the efficacy of SRP in HbA1c% reduction in our pairwise meta-analysis (0.19 [0.08, 0.30]), but not in our network meta-analysis (0.25 [− 0.24, 0.78]). This inconsistency may be attributed to chance alone, as only one study with small sample size provided direct evidence. The inconsistency was also found when comparing SRP with no treatment. SRP could reduce periodontal pathogens, and subsequently inhibit cytokines associated with inflammatory markers, leading to decreased glucose [37]. Our network meta-analysis supported the efficacy of SRP (0.40 [0.088, 0.80]), and was consistent with previous meta-analyses [5, 38]. Although our pairwise meta-analysis showed that SRP was not better than no treatment (0.45 [0.00, 0.89]), the lower bound of the 95% CI was 0. Heterogeneity between SRP and no treatment is extremely high in both pairwise and network meta-analysis, this may due to the fact that the baseline of HbA1c% is not balanced in the 3 included studies and they may have serious methodological issues. For example, they did not report how to perform allocation concealment. More well-executed and multi-center trails are still required to explore the effect of SRP.

We also found an interesting phenomenon that SRP + SDD / local-delivery drugs did not showed any advantages of improving HbA1c% than no treatment. It seems illogical since SRP alone was better than no treatment. There was only one study with small sample sizes in SRP + SDD; further trials are needed to ascertain the role of SDD in improving HbA1c%. The uncomparable baseline of HbA1c% may be attributed to the inefficacy of SRP + local-delivery drugs: HbA1c% > 10 for Santos et al. [30]; HbA1c% < 7 for Pradeep et al. [29], Kumari et al. Sensitivity analyses indicated that the baseline of HbA1c% may influence the efficacy of the periodontal treatments. After we excluded the studies with HbA1c% > 9, the major results did not change greatly. Nonetheless, when the studies with HbA1c% < 7 were excluded, all treatments except SRP + antibiotic could not significantly decrease HbA1c% compared to no treatment. Longitudinal studies involving subjects with different HbA1c% is therefore warranted.

Antibiotics are commonly applied in periodontal treatment. They could significantly decrease the levels of TNF and IL-6 in serum [39]. TNF and IL-6 could impair intracellular insulin signalling, potentially leading to insulin resistance [40]. Contrary to our expectations, we found it couldn’t provide additional benefits compared to SRP alone (0.20 [− 0.17, 0.56]). This was in conformance with findings of Wang et al.(− 0.238 [− 0.616, 0.140]) [11] and Lira Junior et al.(− 0.11[− 0.35,0.13]) [14].

Laser, especially aPDT has been attracting huge interest in adjunct to SRP due to its better antibacterial ability with lower technically sensitive. In our network meta-analysis, we included three RCTs that compared laser to other treatments (SRP + aPDT vs. SRP, SRP + diode laser vs. SRP and SRP + aPDT vs. SRP + Doxy). We found SRP + laser was better compared to no treatment (0.66 [0.097, 1.3]) but not SRP (0.25 [− 0.24, 0.78]). Similarly, a meta-analysis of 2 RCTs was conducted by Abduljabbar et al. showed that aPDT didn’t provide additional benefits compared to SRP alone (0.035 [− 0.47, 0.54]) [13]. However, one of its included RCT [11] was used in treating their patients with doxycycline. In our opinion, it might intensify the effect of aPDT, so we considered it as an independent group (aPDT + Doxy) rather than the laser group. In our network meta-analysis, aPDT + Doxy adjunctive to SRP was ranked best among the comparisons, although there was only one trial of 30 patients was included. It is difficult to draw a solid conclusion with such a small amount of RCTs. Thus, we look forward to more well-executed and multi-center trails.

Regarding FPG, SRP was not better than no treatment (4.91 [− 1.95, 11.78]), and adjuvant treatments did not show any advantage than SRP alone (− 0.28 [− 8.66, 8.11]). The results are consistent with Corbella et al. [17]. However, Sgolastra et al. [41]and Teshome et al. [42] support the effectiveness of periodontal treatment in lowering FPG. Since few studies have reported the results of FPG, it is difficult to draw a conclusion. Therefore, more RCTs were expected to clear out the real effect of periodontal treatments.

This network meta-analysis has several limitations. First, only included English studies were included and their quality is the principal limitation, a low risk of bias was found in six of the 14 included studies. Second, the sample sizes were relatively small, from 30 to 66 subjects. The follow-up duration of the trials was short, resulting in uncertain outcomes. With the foregoing limitations, well-executed and multi-center trails comparing the different adjuvant treatments, and extending the follow-up duration up to 12 or 24 months should be conducted. Finally, this meta-analysis excluded split-mouth RCT, patients with severe T2DM complications and smorkers to meet the transitivity, indicating that the conclusions of this meta-analysis apply to non-smoking CP patients without severe T2DM complications. In addition, though we have adopted strict eligibility criteria, we also found high heterogeneity in comparing SRP with no treatment. We also found a certain publication bias.

## Conclusion

The results of this meta-analysis seem to support that periodontal treatment with aPDT + Doxy possesses the best efficacy in lowering HbA1c% of non-smoking CP without severe T2DM complications. However, the quality of evidence is low or very low, and therefore further studies are needed to confirm the results.

## Additional files

Additional file 1: Search strategy used in PubMed/MEDLINE. (DOCX 14 kb)

Additional file 2: List of excluded duplicate studies. (DOCX 32 kb)

Additional file 3: Sensitivity analysis for informative uniform distribution. (DOCX 24 kb)

Additional file 4: Risk of bias summary: review authors' judgements about each risk of bias item for each included study. (DOCX 342 kb)

Additional file 5: Forest plot of changes in FPG. (DOCX 172 kb)

Additional file 6: Evaluation of model fit. (DOCX 14 kb)

Additional file 7: Sensitivity analysis. (DOCX 16 kb)

Additional file 8: Data extraction table of this network meta-analysis. (DOCX 16 kb)

## Data Availability

All data generated or analysed during this study are included in this published article and Additional file 8.

## Abbreviations

CP: Chronic periodontitis
T2DM: Type 2 diabetes mellitus
SRP: Scaling and root planning
Doxy: Doxycycline
SDD: Subantimicrobial dose doxycycline
SMV: Simvastatin
CHX: Chlorhexidine
aPDT: Antimicrobial photodynamic therapy
AMX: Amoxicillin
MTZ: Metronidazole
ATV: Atorvastatin
DL: Diode laser
PC: Phenothiazine chloride solution
