# Supplementary material for: Effect of non-surgical periodontal therapy on glycemic control of type 2 diabetes mellitus: a systematic review and Bayesian network meta-analysis
Source: BMC Oral Health. 2019 Aug 6;19:176. doi: 10.1186/s12903-019-0829-y (PMC6685286; doi:10.1186/s12903-019-0829-y)
Supplement: Supplementary file 1 — Search strategy used in PubMed/MEDLINE. (DOCX 14 kb) [file 12903_2019_829_MOESM1_ESM.docx]

**Additional file 1**. Search strategy used in PubMed/MEDLINE

|  | Search terms |
| --- | --- |
| No. 5 | No 1 and No 2 and No 3 and No 4 |
| No. 4 | ((randomized controlled trial [Publication Type] OR randomized[Title/Abstract] OR placebo[Title/Abstract])) |
| No. 3 | ((therapy) OR treatment) OR intervention)) |
| No. 2 | ((("Diabetes Mellitus"[Mesh] OR "Diabetes Mellitus, Type 2"[Mesh])) OR (((((((diabetes mellitus, type II[Title/Abstract]) OR type 2 diabetes mellitus[Title/Abstract]) OR T2DM[Text Word]) OR noninsulin dependent diabetes mellitus[Text Word]) OR hyperglycemia[Text Word]) OR glycemic[Text Word]) OR glycosylated hemoglobin[Text Word]))) |
| No. 1 | (((((("Periodontitis"[Mesh] AND "Chronic Periodontitis"[Mesh])) OR (((((adult periodontitis[Title/Abstract]) OR periodontal disease*[Title/Abstract]) OR periodontal pocket[Title/Abstract]) OR attachment loss[Title/Abstract]) OR alveolar bone loss[Title/Abstract]))) |
