# Supplementary material for: Effect of non-surgical periodontal therapy on glycemic control of type 2 diabetes mellitus: a systematic review and Bayesian network meta-analysis
Source: BMC Oral Health. 2019 Aug 6;19:176. doi: 10.1186/s12903-019-0829-y (PMC6685286; doi:10.1186/s12903-019-0829-y)
Supplement: Supplementary file 2 — List of excluded duplicate studies. (DOCX 32 kb) [file 12903_2019_829_MOESM2_ESM.docx]

**Additional file 2**

**List of excluded duplicate studies**

1. Hemoglobin A(1c) Levels Among Patients With Diabetes Receiving Nonsurgical Periodontal Treatment COMMENT & RESPONSE

2. Periodontal treatment and glycemic control in diabetic patients: the problem of a possible Hawthorne effect

3. Analytical epidemiology of periodontitis

4. Antibiotics in aggressive periodontitis, is there a clinical benefit?

5. Antimicrobial photodynamic therapy adjuvant to non-surgical periodontal therapy in patients with diabetes mellitus: A meta-analysis

6. Association Between Periodontal Disease and Erectile Dysfunction: A Systematic Review

7. Association of circulating leptin and adiponectin with periodontitis: a systematic review and meta-analysis

8. Behaviour change counselling for tobacco use cessation and promotion of healthy lifestyles: a systematic review

9. Cinnamon for diabetes mellitus

10. Correlation between periodontal disease management and metabolic control of type 2 diabetes mellitus. A systematic literature review

11. Diabetes and periodontal diseases: interplay and links

12. Diabetes mellitus and periodontal disease

13. Does the adjunctive use of statins provide additional benefits to nonsurgical periodontal treatment? A systematic review and meta-analysis

14. Effect of non-surgical periodontal treatment on glycemic control of patients with diabetes: a meta-analysis of randomized controlled trials

15. Effect of non-surgical periodontal treatment on HbA1c: a meta-analysis of randomized controlled trials[15]

16. Effect of overweight/obesity on response to periodontal treatment: systematic review and a meta-analysis[16]

17. The effect of periodontal therapy on glycemic control and fasting plasma glucose level in type 2 diabetic patients: systematic review and meta-analysis

18. Effect of Periodontal Treatment on Glycemic Control of Diabetic Patients - A systematic review and meta-analysis

19. Effect of periodontal treatment on glycemic control of patients with diabetes: A systematic review and meta-analysis

20. The Effect of Periodontal Treatment on Hemoglobin A1c Levels of Diabetic Patients: A Systematic Review and Meta-Analysis

21. The effect of systemic antibiotics administered during the active phase of non-surgical periodontal therapy or after the healing phase: a systematic review

22. Effectiveness of alendronate as an adjunct to scaling and root planing in the treatment of periodontitis: a meta-analysis of randomized controlled clinical trial

23. Effectiveness of Periodontal Treatment to Improve Metabolic Control in Patients With Chronic Periodontitis and Type 2 Diabetes: A Meta-Analysis of Randomized Clinical Trials

24. Effects of diabetes mellitus on periodontal and peri-implant conditions: update on associations and risks

25. Effects of periodontal therapy on metabolic control in patients with type 2 diabetes mellitus and periodontal disease: a meta-analysis

26. The effects of periodontal treatment on diabetes

27. Effects of periodontal treatment on glycemic control in type 2 diabetic patients: a meta-analysis of randomized controlled trials

28. The effects of periodontal treatment on pregnancy outcomes

29. Effects on HbA1c in diabetic patients of adjunctive use of systemic antibiotics in nonsurgical periodontal treatment: A systematic review

30. Efficacy of bisphosphonate as an adjunct to nonsurgical periodontal therapy in the management of periodontal disease: a systematic review

31. Efficacy of Local Antimicrobials in the Non-Surgical Treatment of Patients With Periodontitis and Diabetes: A Systematic Review

32. Efficacy of periodontal treatment on glycaemic control in diabetic patients: A meta-analysis of interventional studies

33. Efficacy of systemic antibiotics in nonsurgical periodontal therapy for diabetic subjects: a systematic review and meta-analysis

34. Evidence summary: The relationship between oral diseases and diabetes

35. Evidence that periodontal treatment improves diabetes outcomes: a systematic review and meta-analysis

36. Gap Analysis of Older Adults with Type 2 Diabetes Receiving Nonsurgical Periodontal Therapy

37. Influence of obesity on the outcome of non-surgical periodontal therapy - a systematic review

38. Is antimicrobial photodynamic therapy an effective treatment for chronic periodontitis in diabetes mellitus and cigarette smokers: a systematic review and meta-analysis

39. Management of diabolical diabetes mellitus and periodontitis nexus: Are we doing enough?

40. The Multiple Roles of Vitamin D in Human Health. A Mini-Review

41. Non-antibacterial tetracycline formulations: host-modulators in the treatment of periodontitis and relevant systemic diseases

42. Oral inflammation and infection, and chronic medical diseases: implications for the elderly

43. An overview of systematic reviews on the effectiveness of periodontal treatment to improve glycaemic control

44. Periodontal disease and glycemic control in diabetics

45. Periodontal Therapy and Systemic Inflammation in Type 2 Diabetes Mellitus: A Meta-Analysis

46. Periodontal treatment and glycaemic control in patients with diabetes and periodontitis: an umbrella review

47. Periodontal treatment could improve glycaemic control in diabetic patients

48. Periodontal treatment: Potential to reduce cardiovascular morbidity and/or mortality

49. Periodontitis and systemic diseases: a record of discussions of working group 4 of the Joint EFP/AAP Workshop on Periodontitis and Systemic Diseases

50. Prevalence of Peri-implantitis in Medically Compromised Patients and Smokers: A Systematic Review

51. Review of Motivational Interviewing in promoting health behaviors

52. Role of Dentifrices With Essential Oil Formulations in Periodontal Healing

53. Role of lasers as an adjunct to scaling and root planing in patients with type 2 diabetes mellitus: a systematic review

54. Scaling and Root-planing (SRP) May Improve Glycemic Control and Lipid Profile in Patients With Chronic Periodontitis (CP) and Type 2 Diabetes (DM2) in a Specific Subgroup: A Meta-analysis of Randomized Clinical Trials

55. Specialized proresolving mediator targets for RvE1 and RvD1 in peripheral blood and mechanisms of resolution

56. Statins, bone metabolism and treatment of bone catabolic diseases

57. Systematic review and assessment of systematic reviews examining the effect of periodontal treatment on glycemic control in patients with diabetes

58. Systemic antimicrobials adjuvant to periodontal therapy in diabetic subjects: a meta-analysis

59. Treating periodontal disease may improve metabolic control in diabetics

60. Treatment of Class II Molar Furcation Involvement: Meta-Analyses of Reentry Results

61. Treatment of periodontal disease for glycaemic control in people with diabetes

62. Treatment of periodontal disease for glycaemic control in people with diabetes mellitus

63. Treatment of periodontitis improves the atherosclerotic profile: a systematic review and meta-analysis

64. Using probiotics in clinical practice: Where are we now? A review of existing meta-analyses

65. Weak Evidence Hinders the Understanding of the Benefits of Periodontal Therapy on Glycemic Control in Patients with Diabetes and Periodontitis

66. Why Should Patients with Systemic Disease and Tobacco Smokers Go to the Dentist?

67. LASER curettage as adjunct to SRP, compared to SRP alone, in patients with periodontitis and controlled type 2 diabetes mellitus: a comparative clinical study

68. Local adjunct effect of antimicrobial photodynamic therapy for the treatment of chronic periodontitis in type 2 diabetics: split-mouth double-blind randomized controlled clinical trial

69. Role of Low-Level Laser Therapy as an Adjunct to Initial Periodontal Treatment in Type 2 Diabetic Patients: a Split-Mouth, Randomized, Controlled Clinical Trial

70. 0.1% chlorhexidine mouthwash for gingival inflammation in diabetic adults: double blind study

71. Adjunctive use of modified Yunu-Jian in the non-surgical treatment of male smokers with chronic periodontitis: a randomized double-blind, placebo-controlled clinical trial

72. Calcineurin inhibitors in renal transplantation: What is the best option?

73. Cardiovascular disease in autoimmune rheumatic diseases

74. Changes in inflammation and bone turnover markers after treatment for periodontal disease in patients with diabetes

75. Changes in inflammatory mediators in gingival crevicular fluid following periodontal disease treatment in pregnancy: relationship to adverse pregnancy outcome

76. Clinical trial of the effects of vitamin D supplementation on psychological symptoms and metabolic profiles in maintenance methadone treatment patients

77. Combination of everolimus with trastuzumab plus paclitaxel as first-line treatment for patients with HER2-positive advanced breast cancer (BOLERO-1): A phase 3, randomised, double-blind, multicentre trial

78. Comparative evaluation of adjunctive oral irrigation in diabetics

79. Comparative risk of chronic kidney diseases in patients with urolithiasis and urological interventions: a longitudinal population-based study

80. Current Medical Therapy for Chronic Inflammatory Bowel Diseases

81. Dietary Carbohydrates and Dental-Systemic Diseases

82. Double-blinded study to investigate effectiveness of 980nm diode laser for chronic periodontal disease-preliminary results

83. Effect of periodontal disease and non surgical periodontal treatment on C-reactive protein. Evaluation of type 1 diabetic patients

84. Effect of basic periodontal treatment on glycemic control and inflammation in patients with diabetes mellitus type 1 and type 2: controlled clinical trial

85. The effect of long-term aspirin intake on the outcome of non-surgical periodontal therapy in smokers: a double-blind, randomized pilot study

86. Effect of Nd:YAG Laser-Assisted Non-Surgical Periodontal Therapy on Clinical Periodontal and Serum Biomarkers in Patients With and Without Coronary Artery Disease: A Short-Term Pilot Study

87. Effect of Non-Surgical Periodontal Therapy Along With Myo-Inositol on High-Sensitivity C-Reactive Protein and Insulin Resistance in Women With Polycystic Ovary Syndrome and Chronic Periodontitis: A Randomized Controlled Trial

88. Effect of non-surgical periodontal treatment with or without doxycycline on the periodontium of type 1 diabetic patients

89. Effect of periodontal disease and non surgical periodontal treatment on C-reactive protein. Evaluation of type 1 diabetic patients

90. Effect of Periodontal Therapy on Arterial Structure and Function Among Aboriginal Australians A Randomized, Controlled Trial

91. The effect of periodontal therapy on cardiovascular risk markers: a 6-month randomized clinical trial

92. Effect of periodontal therapy with systemic antimicrobials on parameters of metabolic syndrome: A randomized clinical trial

93. The effect of periodontal treatment on metabolic control of type 1 diabetes mellitus

94. Effect of periodontal treatment on the clinical parameters of patients with rheumatoid arthritis: study protocol of the randomized, controlled ESPERA trial

95. Effect of sustained PDGF nonviral gene delivery on repair of tooth-supporting bone defects

96. Effectiveness of the topical application of fluor on the moderate gingivitis in diabetic type 2

97. Effects of full-mouth scaling on the periodontal health of Indigenous Australians: a randomized controlled trial

98. Effects of ketorolac tromethamine mouthrinse (0.1%) on crevicular fluid prostaglandin E2 concentrations in untreated chronic periodontitis

99. Effects of Standardised Fermented Papaya Gel on Clinical Symptoms, Inflammatory Cytokines, and Nitric Oxide Metabolites in Patients with Chronic Periodontitis: An Open Randomised Clinical Study

100. Efficacy and safety of once-daily QVA149 compared with twice-daily salmeterol-fluticasone in patients with chronic obstructive pulmonary disease (ILLUMINATE): A randomised, double-blind, parallel group study

101. Efficacy of locally-delivered 1% metformin gel in the treatment of intrabony defects in patients with chronic periodontitis: a randomized, controlled clinical trial

102. Efficacy of scaling and root planning with and without adjunct Nd:YAG laser therapy on clinical periodontal parameters and gingival crevicular fluid interleukin 1-beta and tumor necrosis factor-alpha levels among patients with periodontal disease: A prospective randomized split-mouth clinical study

103. European Guidelines on cardiovascular disease prevention in clinical practice (version 2012)

104. Evaluating Periodontal Treatment to Prevent Cardiovascular Disease: challenges and Possible Solutions

105. Evaluation of non-surgical periodontal treatment in patients with a past history of myocardial infarction

106. Evaluation of the effect of professional dental cleaning and education in dental hygiene in type 1 diabetic patients

107. Everolimus for advanced pancreatic neuroendocrine tumours: A subgroup analysis evaluating japanese patients in the radiant-3 trial

108. Factors affecting the periapical healing process of endodontically treated teeth

109. Full-mouth tooth extraction lowers systemic inflammatory and thrombotic markers of cardiovascular risk

110. Healing response to non-surgical periodontal therapy in patients with diabetes mellitus: clinical, microbiological, and immunologic results

111. The Influence of Triclosan on Biomarkers of Cardiovascular Risk in Patients in the Cardiovascular and Periodontal Study (CAPS): a Randomized Controlled Trial

112. Integrative selection of oral hygiene complex for pregnant women with diabetes mellitus and periodontal disease

113. The Kidney and Periodontal Disease (KAPD) study: a pilot randomized controlled trial testing the effect of non-surgical periodontal therapy on chronic kidney disease

114. Lactobacillus reuteri oral administration improves periodontal disease in children and adolescents with type 1 diabetes

115. Locally delivered 1% metformin gel in the treatment of smokers with chronic periodontitis: a randomized controlled clinical trial

116. Long-term control of diabetes mellitus and periodontitis

117. Long-term safety and efficacy of TAK-085 in Japanese subjects with hypertriglyceridemia undergoing lifestyle modification: the omega-3 fatty acids randomized long-term (ORL) study

118. Long-term use of medications and destructive periodontal disease

119. Major Adverse Cardiovascular Events in Treated Periodontitis: a Population-Based Follow-Up Study from Taiwan

120. Marginal bone stability using 3 different flap approaches for alveolar split expansion for dental implants: a 1-year clinical study

121. Mechanisms and therapeutic effectiveness of lactobacilli

122. Multicenter, open-label phase i clinical study of oral rigosertib in japanese patients with recurrent/relapsed or refractory myelodysplastic syndromes: tolerability, efficacy, and pharmacokinetic profiles

123. Oral administration of lactobacillus reuteri improves periodontal disease in children and adolescents with type 1 diabetes

124. Oral disease and subsequent cardiovascular disease in people with type 2 diabetes: a prospective cohort study based on the Action in Diabetes and Vascular Disease: Preterax and Diamicron Modified-Release Controlled Evaluation (ADVANCE) trial

125. An oral health optimized diet can reduce gingival and periodontal inflammation in humans - a randomized controlled pilot study

126. Periodontal Disease and Atherosclerotic Vascular Disease: Does the Evidence Support an Independent Association? A Scientific Statement From the American Heart Association

127. Periodontal disease and chronic kidney disease among Aboriginal adults; An RCT

128. Periodontal disease and type 1 diabetes mellitus: associations with glycemic control and complications: an Indian perspective

129. Periodontal treatment by Arestin and its effects on glycemic control in type 1 diabetes patients

130. Potential Role of Free Fatty Acids in the Pathogenesis of Periodontitis and Primary Sjogren's Syndrome

131. Prevalence and risk factors of periodontal disease among pre-conception Chinese women

132. Prevention of cardiovascular disease in rheumatoid arthritis

133. Professional oral hygiene procedures do not influence plasma glucose levels in systemically healthy individuals: A short-term, randomised, controlled trial

134. A randomized controlled trial of pre-conception treatment for periodontal disease to improve periodontal status during pregnancy and birth outcomes

135. A randomized phase II study of everolimus for advanced pancreatic neuroendocrine tumors in Chinese patients

136. Rapid carotid plaques reversal of PCSK9 inhibitors when added to statins and eicosapentaenoic acid in high risk cardiovascular patients

137. Role of mechanical curettage with and without adjunct antimicrobial photodynamic therapy in the treatment of peri-implant mucositis in cigarette smokers: A randomized controlled clinical trial

138. Safety, tolerability, and pharmacokinetics of radavirsen (AVI-7100), an antisense oligonucleotide targeting influenza a M1/M2 translation

139. Secondary prevention of coronary artery disease with antimicrobials: current status and future directions

140. Single-blind studies of the effects of improved periodontal health on metabolic control in type 1 diabetes mellitus

141. Surgical and non-surgical therapy with systemic antimicrobials for residual pockets in type 2 diabetics with chronic periodontitis: a pilot study

142. The Tennessee study: factors affecting treatment outcome and healing time following nonsurgical root canal treatment

143. Therapy with adjunctive doxycycline local delivery in patients with type 1 diabetes mellitus and periodontitis

144. Trial testing the impact of professional oral hygiene and patient oral hygiene education on the parodontal status in type 1 diabetic patients

145. Use of antiseptic mouthrinse during pregnancy and pregnancy outcomes: a randomised controlled clinical trial in rural China

146. Adverse events associated with chlorhexidine use: results from the Department of Veterans Affairs Dental Diabetes Study

147. Antimicrobial photodynamic therapy combined with periodontal treatment for metabolic control in patients with type 2 diabetes mellitus: study protocol for a randomized controlled trial

148. Commentary: Periodontal Treatment and Inflammation in Diabetes: Association or Causation?

149. Diabetes and periodontal diseases: consensus report of the Joint EFP/AAP Workshop on Periodontitis and Systemic Diseases

150. Diabetes and periodontal therapy

151. Diabetes Mellitus and Periodontal Diseases

152. Effect of gingival application of melatonin on alkaline and acid phosphatase, osteopontin and osteocalcin in patients with diabetes and periodontal disease

153. The effect of intensive oral hygiene care on gingivitis and periodontal destruction in type 2 diabetic patients

154. Effect of nonsurgical periodontal therapy on insulin resistance in patients with type 2 diabetes mellitus and chronic periodontitis

155. The effect of oral hygiene instructions on diabetic type 2 male patients with periodontal diseases

156. Effects of periodontal therapy on glucose management in people with diabetes mellitus

157. Evaluation of the relationship between type 2 diabetes and periodontal disease

158. Exodontia may improve glycemic control of diabetic patients with periodontitis

159. Guidelines for the management of pemphigus vulgaris

160. Human oral microbiota and its modulation for oral health

161. Influence of Body Mass Index and Other Periodontitis-associated Risk Factors and Risk Indicators on Periodontal Treatment Needs: A Cross-sectional Study

162. Is a high level of total cholesterol a risk factor for dental implants or bone grafting failure? A retrospective cohort study on 227 patients

163. Mitochondrial oxidative stress, inflammation and endothelial function in people with type 2 diabetes

164. The multi-center randomized controlled trial (RCT) published by the journal of the American Medical Association (JAMA) on the effect of periodontal therapy on glycated hemoglobin (HbA1c) has fundamental problems

165. Non-surgical periodontal therapy and serum lipid levels in patients with diabetes mellitus

166. Non-surgical periodontal treatment and lipid levels in diabetic patients

167. Nutrigenomics: the role of nutrients in gene expression

168. Nutrition and health: Guidelines for dental practitioners

169. Periodontal and glycemic effects of nonsurgical periodontal therapy in patients with type 2 diabetes stratified by baseline HbA1c

170. Periodontal conditions in a group of Colombian type 2 diabetic patients with different degrees of metabolic control

171. Periodontal disease ñ a further potentially modifiable risk factor limiting conception ñ a case for a pre-pregnancy dental check-up?

172. Periodontal disease related to diabetic status. A pilot study of the response to periodontal therapy in type 1 diabetes

173. Position of the American Dietetic association: Oral health and nutrition

174. Relationship Among Mothers' Glycemic Level, Periodontitis, and Birth Weight

175. Relationship between diabetes and periodontal infection

176. Risk assessment in clinical practice

177. Routine prophylaxes every 3 months improves chronic periodontitis status in type 2 diabetes

178. Short-term effects of non-surgical periodontal therapy on clinical measures of impaired glucose tolerance in people with prediabetes and chronic periodontitis

179. Studies on periodontitis and analyses of individuals at risk for periodontal diseases

180. Systemic Propolis (Adjuvant to Nonsurgical Periodontal Treatment) May Aid in Glycemic Control and Periodontal Health in Type 2 Diabetes of Long Duration

181. Treatment of gingivitis and periodontal diseases with insadol in diabetics

182. Two faces of microbiota in inflammatory and autoimmune diseases: triggers and drugs

183. Type 2 diabetes and oral health: a comparison between diabetic and non-diabetic subjects

184. Update on diabetes mellitus: prevention, treatment, and association with oral diseases

185. Vitamin D Earns More than a Passing Grade

186. 25-Hydroxyvitamin D-3-Loaded PLA Microspheres: In Vitro Characterization and Application in Diabetic Periodontitis Models

187. Chronic treatment with the glucocorticoid receptor antagonist RU486 inhibits diabetes-induced enhancement of experimental periodontitis

188. High-fat diet induces periodontitis in mice through lipopolysaccharides (LPS) receptor signaling: protective action of estrogens

189. Histometric Analysis of the Effect of Enamel Matrix Derivative on the Healing of Periodontal Defects in Rats With Diabetes

190. Intermittent administration of parathyroid hormone ameliorated alveolar bone loss in experimental periodontitis in streptozotocin-induced diabetic rats

191. Clinical and metabolic changes after conventional treatment of type 2 diabetic patients with chronic periodontitis

192. Clinical and metabolic evaluation of one-stage, full-mouth, ultrasonic debridement as a therapeutic approach for uncontrolled type 2 diabetic patients with periodontitis

193. Effect of maintenance therapy with or without the use of chlorhexidine in teeth restored with composite resin in patients with diabetes mellitus

194. Effects of saliva substitutes on oral status in patients with Type 2 diabetes

195. Efficacy and safety of avandamet or uptitrated metformin treatment in patients with type 2 diabetes inadequately controlled with metformin alone: a multicenter, randomized, controlled trial

196. Efficacy and safety of canagliflozin in Japanese patients with type 2 diabetes: a randomized, double-blind, placebo-controlled, 12-week study

197. Efficacy and safety of luseogliflozin monotherapy in Japanese patients with type 2 diabetes mellitus: a 12-week, randomized, placebo-controlled, phase II study

198. Efficacy of non-surgical periodontal therapy with adjunct Nd:YAG laser therapy in the treatment of periodontal inflammation among patients with and without type 2 diabetes mellitus: A short-term pilot study

199. Glycated hemoglobin improvement by oral appliance therapy in obstructive sleep apnea syndrome patients with diabetes mellitus

200. Green tea extract as a local drug therapy on periodontitis patients with diabetes mellitus: A randomized case-control study

201. Influence of Glycemic Control on Peri-Implant Bone Healing: 12-Month Outcomes of Local Release of Bone-Related Factors and Implant Stabilization in Type 2 Diabetics

202. Low-level lasers as an adjunct in periodontal therapy in patients with diabetes mellitus

203. Ozontherapy in diabetic patients with periodontal diseases, Ozonoterapia en pacientes diabioticos con enfermedad periodontal

204. Peri-implant evaluation of immediately loaded implants placed in esthetic zone in patients with diabetes mellitus type 2: a two-year study

205. Peri-implant Tissues and Diseases

206. A Prospective Case-Control Clinical Study of Titanium-Zirconium Alloy Implants with a Hydrophilic Surface in Patients with Type 2 Diabetes Mellitus

207. A short-term evaluation of the relationship between plasma ascorbic acid levels and periodontal disease in systemically healthy and type 2 diabetes mellitus subjects

208. Sitagliptin added to treatment with ongoing pioglitazone for up to 52 weeks improves glycemic control in Japanese patients with type 2 diabetes

209. The effect of full-mouth tooth extraction on glycemic control among patients with type 2 diabetes requiring extraction of all remaining teeth: a randomized clinical trial

210. The Effect of Resin Composites and Polishing Procedure on Periodontal Tissue Parameters in Patients with Diabetes Mellitus

211. Effectiveness of lifestyle change plus dental care program in improving glycemic and periodontal status in aging patients with diabetes: a cluster, randomized, controlled trial

212. Estimation of gingival crevicular blood glucose level for the screening of diabetes mellitus: A simple yet reliable method

213. Glucose-lowering effects and safety of DS-8500a, a G protein-coupled receptor 119 agonist, in japanese patients with type 2 diabetes: Results of a randomized, double-blind, placebo-controlled, parallel-group, multicenter, phase II study

214. Health promotion for patients with diabetes: health coaching or formal health education?

215. Impact of Diabetes Mellitus on Intravascular Ultrasound-Guided Provisional Stenting in Coronary Bifurcation Lesions J-REVERSE Sub-Study

216. The Impact of Resveratrol Supplementation on Blood Glucose, Insulin, Insulin Resistance, Triglyceride, and Periodontal Markers in Type 2 Diabetic Patients with Chronic Periodontitis

217. Local levels of biomarkers after surgical and nonsurgical debridement of residual pockets and nonresidual sites in diabetic patients: a 12-month follow-up

218. A randomized clinical trial comparing the efficacy of mandibular implant-supported overdentures and conventional dentures in diabetic patients. Part I: methodology and clinical outcomes

219. Six-month outcomes in dental patients identified with hyperglycaemia: a randomized clinical trial

220. Unsplinted implants retaining a mandibular overdenture with magnetic attachments: A short-term clinical study in controlled type 2 diabetic patients

221. The effect of antimicrobial periodontal therapy on type 2 diabetes mellitus. (Annual meeting Abstract)

222. Translational Science 2012 Meeting Abstracts, Washington, DC, USA, April 18-20, 2012 Abstracts

223. Effect of nonsurgical periodontal therapy (with or without oral doxycycline delivery) on glycemic status and clinical periodontal parameters in patients with prediabetes: a short-term longitudinal randomized case-control study

224. Impact of scaling and root planing on clinical periodontal status and glycemic levels in prediabetic patients

225. Additional effects of aPDT on nonsurgical periodontal treatment with doxycycline in type II diabetes: A randomized, controlled clinical trial

226. Adjunctive Local Delivery of Aloe Vera Gel in Patients With Type 2 Diabetes and Chronic Periodontitis: a Randomized, Controlled Clinical Trial

227. Amoxicillin Plus Metronidazole Therapy for Patients with Periodontitis and Type 2 Diabetes: a 2-year Randomized Controlled Trial

228. Antimicrobial photodynamic therapy as an alternative to systemic antibiotics: results from a double-blind, randomized, placebo-controlled, clinical study on type 2 diabetics

229. Effect of non-surgical periodontal treatment on visfatin concentrations in serum and gingival crevicular fluid of patients with chronic periodontitis and type 2 diabetes mellitus

230. The effect of periodontal therapy on the improvement of glycemic control in patients with type 2 diabetes mellitus: A randomized controlled clinical trial

231. Effect of photodynamic therapy as an adjuvant to non-surgical periodontal therapy: periodontal and metabolic evaluation in patients with type 2 diabetes mellitus

232. Effect of scaling and root planing combined with systemic doxycycline therapy on glycemic control in diabetes mellitus subjects with chronic generalized periodontitis: a clinical study

233. Effects of doxycycline on clinical, microbiological and immunological parameters in well-controlled diabetes type-2 patients with periodontal disease: a randomized, controlled clinical trial

234. Efficacy of nonsurgical periodontal therapy on glycaemic control in type II diabetic patients: a randomized controlled clinical trial

235. Efficacy of short-term adjunctive subantimicrobial dose doxycycline in diabetic patients - randomized study

236. Efficacy of Subgingivally Delivered 1.2% Atorvastatin in the Treatment of Chronic Periodontitis in Patients With Type 2 Diabetes Mellitus: A Randomized Controlled Clinical Trial

237. Efficacy of subgingivally delivered simvastatin in the treatment of patients with type 2 diabetes and chronic periodontitis: a randomized double-masked controlled clinical trial

238. Full-mouth disinfection as a therapeutic protocol for type-2 diabetic subjects with chronic periodontitis: twelve-month clinical outcomes: a randomized controlled clinical trial

239. Locally Delivered 0.5% Azithromycin as an Adjunct to Non-Surgical Treatment in Patients With Chronic Periodontitis With Type 2 Diabetes: A Randomized Controlled Clinical Trial

240. Locally delivered 0.5% clarithromycin, as an adjunct to nonsurgical treatment in chronic periodontitis with well-controlled type 2 diabetes: a randomized controlled clinical trial

241. Metronidazole and amoxicillin as adjuncts to scaling and root planing for the treatment of type 2 diabetic subjects with periodontitis: 1-year outcomes of a randomized placebo-controlled clinical trial

242. Non-surgical periodontal therapy affects metabolic control in diabetics: a randomized controlled clinical trial

243. Nonsurgical periodontal therapy with/without diode laser modulates metabolic control of type 2 diabetics with periodontitis: a randomized clinical trial

244. Periodontal Treatment and Inflammation in Diabetes: Association or Causation?

245. Alterations in HbA1c following minimal or enhanced non-surgical, non-antibiotic treatment of gingivitis or mild periodontitis in type 2 diabetic patients: a pilot trial

246. Benefits of non-surgical periodontal treatment in patients with type 2 diabetes mellitus and chronic periodontitis: A randomized controlled trial

247. Changes in crevicular cytokines after application of melatonin in patients with periodontal disease

248. Changes in diabetes medications in the Diabetes and Periodontal Therapy Trial and their effect on hemoglobin A1c (HbA1c)

249. Changes of adiponectin and inflammatory cytokines after periodontal intervention in type 2 diabetes patients with periodontitis

250. Clinical and radiological improvement of periodontal disease in patients with type 2 diabetes mellitus treated with alendronate: a randomized, placebo-controlled trial

251. Design features of the Diabetes and Periodontal Therapy Trial (DPTT): a multicenter randomized single-masked clinical trial testing the effect of nonsurgical periodontal therapy on glycosylated hemoglobin (HbA1c) levels in subjects with type 2 diabetes and chronic periodontitis

252. Does periodontal care improve glycemic control? The Department of Veterans Affairs Dental Diabetes Study

253. Effect of antimicrobial periodontal treatment and maintenance on serum adiponectin in type 2 diabetes mellitus

254. Effect of community periodontal care intervention on periodontal health and glycemic control in type 2 diabetic patients with chronic periodontitis

255. The effect of improved periodontal health on metabolic control in type 2 diabetes mellitus

256. Effect of non-invasive periodontal therapy on glycaemic control in type 2 diabetes mellitus patients - A randomized control trial

257. Effect of non-surgical periodontal therapy on C-reactive protein, oxidative stress, and matrix metalloproteinase (MMP)-9 and MMP-2 levels in patients with type 2 diabetes: a randomized controlled study

258. Effect of non-surgical periodontal therapy on glycemic control in patients with type 2 diabetes mellitus

259. The effect of nonsurgical periodontal therapy on hemoglobin A1c levels in persons with type 2 diabetes and chronic periodontitis: a randomized clinical trial

260. Effect of non-surgical periodontal therapy on insulin resistance in patients with type II diabetes mellitus and chronic periodontitis, as assessed by C-peptide and the Homeostasis Assessment Index

261. Effect of non-surgical periodontal therapy on patients with type 2 diabetes mellitus

262. Effect of nonsurgical periodontal therapy verses oral hygiene instructions on type 2 diabetes subjects with chronic periodontitis: a randomised clinical trial

263. Effect of omega 3 fatty acids plus low-dose aspirin on both clinical and biochemical profiles of patients with chronic periodontitis and type 2 diabetes: a randomized double blind placebo-controlled study

264. The effect of periodontal therapy on glycaemic control in a Hispanic population with type 2 diabetes: a randomized controlled trial

265. Effect of periodontal therapy on glycemic control and circulating TNF-alpha in type 2 diabetic patients

266. The effect of periodontal therapy on uncontrolled type 2 diabetes mellitus in older subjects

267. Effect of scaling and root planing with and without adjunctive use of an essential-oil-based oral rinse in the treatment of periodontal inflammation in type-2 diabetic patients

268. Effectiveness of full-mouth and partial-mouth scaling and root planing in treating chronic periodontitis in subjects with type 2 diabetes

269. Effectiveness of lifestyle change plus dental care (LCDC) program on improving glycemic and periodontal status in the elderly with type 2 diabetes

270. Effects of nonsurgical periodontal therapy in patients with moderately controlled type 2 diabetes mellitus and chronic periodontitis in Nepalese population

271. Effects of non-surgical periodontal treatment on clinical response, serum inflammatory parameters, and metabolic control in patients with type 2 diabetes: a randomized study

272. Effects of nonsurgical periodontal treatment on glycated haemoglobin on type 2 diabetes patients (PARODIA 1 study): a randomized controlled trial in a sub-Saharan Africa population

273. The effects of non-surgical periodontal treatment on glycemic control, oxidative stress balance and quality of life in patients with type 2 diabetes: a randomized clinical trial

274. Effects of periodontal non-surgical therapy plus azithromycin on glycemic control in patients with diabetes: a randomized clinical trial

275. Effects of periodontal therapy on glycemic control and inflammatory markers

276. Efficacy of Subgingivally Delivered Satranidazole in the Treatment of Type 2 Diabetes Subjects with Chronic Periodontitis: A Randomized Controlled Clinical Trial

277. Efficacy of systemic administration of alpha lipoic acid and scaling and root planning in patients with chronic periodontitis and type 2 diabetes mellitus-A randomised controlled trial

278. Evaluation of adjunctive systemic doxycycline with nonsurgical periodontal therapy within type 2 diabetic patients

279. Evaluation of different periodontal treatments modes for patients with type 2 diabetes mellitus

280. Evaluation of subantimicrobial dose doxycycline as an adjunct to scaling and root planing in chronic periodontitis patients with diabetes: a randomized, placebo-controlled clinical trial

281. Factors associated with the clinical response to nonsurgical periodontal therapy in people with type 2 diabetes mellitus

282. Full-mouth scaling and root planing in type 2 diabetic subjects: one-year microbiological outcomes

283. Glycemic control and adipokines after periodontal therapy in patients with Type 2 diabetes and chronic periodontitis

284. Impact of combined non-surgical and surgical periodontal treatment in patients with type 2 diabetes mellitus-a preliminary report randomized clinical study

285. Impact of cranberry juice enriched with omega-3 and their individual consumption adjunct with nonsurgical periodontal treatment on glycemic control and lipid profile in patients with diabetes mellitus and periodontal disease

286. Impact of Cranberry Juice Enriched with Omega-3 Fatty Acids Adjunct with Nonsurgical Periodontal Treatment on Metabolic Control and Periodontal Status in Type 2 Patients with Diabetes with Periodontal Disease

287. The impact of resveratrol supplementation on blood glucose, insulin, insulin resistance, triglyceride and periodontal markers in type 2 diabetic patients with chronic periodontitis

288. Improvement of glycated hemoglobin in Japanese subjects with type 2 diabetes by resolution of periodontal inflammation using adjunct topical antibiotics: results from the Hiroshima Study

289. Inflammatory cytokines, adiponectin, insulin resistance and metabolic control after periodontal intervention in patients with type 2 diabetes and chronic periodontitis

290. Local drug delivery of alendronate gel for the treatment of patients with chronic periodontitis with diabetes mellitus: a double-masked controlled clinical trial

291. Microbiological effects of periodontal therapy plus azithromycin in patients with diabetes: results from a randomized clinical trial

292. Non-surgical periodontal therapy with and without subgingival minocycline administration in patients with poorly controlled type II diabetes: a randomized controlled clinical trial

293. Partial- and full-mouth scaling and root planing in type 2 diabetic subjects: a 12-mo follow-up of clinical parameters and levels of cytokines and osteoclastogenesis-related factors

294. Periodontal therapy and glycaemic control among individuals with type 2 diabetes: reflections from the PerioCardio study

295. Periodontal treatment to improve glycaemic control in diabetic patients: study protocol of the randomized, controlled DIAPERIO trial

296. Periodontal treatment with topical antibiotics improves glycemic control in association with elevated serum adiponectin in patients with type 2 diabetes mellitus

297. Propolis Improves Periodontal Status and Glycemic Control in Patients With Type 2 Diabetes Mellitus and Chronic Periodontitis: A Randomized Clinical Trial

298. A randomized, controlled trial on the effect of non-surgical periodontal therapy in patients with type 2 diabetes. Part I: effect on periodontal status and glycaemic control

299. The role of self-efficacy in health coaching and health education for patients with type 2 diabetes

300. Scaling and root planing with enhanced root planing on healthcare for type 2 diabetes mellitus: A randomized controlled clinical trial

301. Short-term effects of 2% atorvastatin dentifrice as an adjunct to periodontal therapy: a randomized double-masked clinical trial

302. Short-term effects of photodynamic therapy on periodontal status and glycemic control of patients with diabetes

303. Study design, recruitment, and baseline characteristics: the Department of Veterans Affairs Dental Diabetes Study

304. Sub-antimicrobial doxycycline for periodontitis reduces hemoglobin A1c in subjects with type 2 diabetes: a pilot study

305. Supragingival biofilm control and systemic inflammation in patients with type 2 diabetes mellitus

306. Systemic Inflammatory Biomarkers and Their Association With Periodontal and Diabetes-Related Factors in the Diabetes and Periodontal Therapy Trial, A Randomized Controlled Trial

307. Systemic lycopene as an adjunct to scaling and root planing in chronic periodontitis patients with type 2 diabetes mellitus

308. Treatment of periodontal disease in diabetics reduces glycated hemoglobin

309. Local levels of biomarkers after surgical and nonsurgical debridement of residual pockets and nonresidual sites in diabetic patients: a 12-month follow-up

310. Evaluation of first phase non-surgical periodontal therapy on diabetes control

311. Clinical, microbial, and immune responses observed in patients with diabetes after treatment for gingivitis: a three-month randomized clinical trial

312. Impact of gingivitis treatment for diabetic patients on quality of life related to periodontal objective parameters: A randomized controlled clinical trial

313. Oral probiotics in the management of gingivitis in diabetic patients: a double blinded randomized controlled study

314. A Traditional Mouthwash (Punica granatum var pleniflora) for Controlling Gingivitis of Diabetic Patients: A Double-Blind Randomized Controlled Clinical Trial

315. The effect of initial periodontal therapy on metabolic control in type 2 diabetes mellitus

316. Efficacy of short-term adjunctive subantimicrobial dose doxycycline in diabetic patients--randomized study

317. Association of glycaemia with macrovascular and microvascular complications of type 2 diabetes (UKPDS 35): prospective observational study
