# Supplementary material for: Effect of non-surgical periodontal therapy on glycemic control of type 2 diabetes mellitus: a systematic review and Bayesian network meta-analysis
Source: BMC Oral Health. 2019 Aug 6;19:176. doi: 10.1186/s12903-019-0829-y (PMC6685286; doi:10.1186/s12903-019-0829-y)
Supplement: Supplementary file 3 — Sensitivity analysis for informative uniform distribution. (DOCX 24 kb) [file 12903_2019_829_MOESM3_ESM.docx]

| Additional file 3. Risk of bias summary: review authors' judgements about each risk of bias item for each included study. | | | | | | | |
| --- | --- | --- | --- | --- | --- | --- | --- |
| First author,  year | Random sequence generation | Allocation concealment | Blinding of participants and personnel | Incomplete outcome data | Selective outcome reporting | Other bias | Overall risk of bias |
| Singh (2008) | Unclear - unclear information on random sequence generation: “The selected patients were randomly assigned to three groups …” | Unclear – no information provided | High risk - no placebos used | Low risk – no drop-outs reported | Unclear - no obvious selective reporting; it is difficult to judge however whether selective reporting is a problem, as no protocol exists | Unclear - unknown if baseline imbalances between groups | High risk |
| Gilowski (2012) | Low risk – “patients were randomly (block randomization method) assigned to one of the study group…” | Unclear – no information provided | Low risk – “Only the study statisticians and the data monitoring committee saw unblinded data, but none had any contact with study participants” | Unclear - Insufficient information to judge the numbers used in each analysis | High risk - not reported CAL data although measured | Low risk - no other sources | High risk |
| Moeintaghavi (2012) | Low risk – “… the study research  assistant (KK) using a computer generated random  numbers table” | Unclear – no information provided | High risk - not possible | Low risk – no drop-outs reported | High risk - adverse events not reported | Low risk - no other sources | High risk |
| Gaikwad (2013) | Unclear - unclear information on random sequence generation: “The selected subjects were randomly assigned to two groups …” | Unclear – no information provided | High risk - no placebos used | Low risk – low drop-out rate (16%), 8 subjects lost, while within-person design ensures balance across randomization groups. | Unclear – no obvious selective reporting; it is difficult to judge however whether selective reporting is a problem, as no protocol exists. | Low risk - no other sources | High risk |
| Pradeep (2013) | Low risk – “The randomization process  was made externally by the statistical unit using a computer-generated random table” | Low risk – “investigators  were neither involved in the randomization process nor were they aware of the assigned group in all outcome evaluations” | Unclear - not report if blinding of clinical operator | Low risk – low drop-out rate (7.9%), while within-person design ensures balance across randomization groups. | Unclear – no obvious selective reporting; it is difficult to judge however whether selective reporting is a problem, as no protocol exists. | Low risk - no other sources | Unclear |
| Santos (2013) | Low risk – “The study coordinator (PMD) used a  computer-generated table to randomly allocate the subjects…” | Low risk – “Identical plastic bottles containing the placebo or CHX gels and rinses were sent to the study coordinator, who marked the code number of each subject on each bottle” | Unclear – “the examiner could deduce the subjects that were receiving CHX or placebo solutions”, but the study state this problem in its manuscript. | Low risk – low drop-out rate (13.1%), while within-person design ensures balance across randomization groups. | Unclear – no obvious selective reporting; it is difficult to judge however whether selective reporting is a problem, as no protocol exists. | Low risk - no other sources | Unclear |
| Telgi (2013) | Unclear - unclear information on random sequence generation: “All of the 60 patients were randomly divided into 3 equal groups” | Unclear – no information provided | High risk - not possible | Low risk – no drop-outs reported | Unclear – no obvious selective reporting; it is difficult to judge however whether selective reporting is a problem, as no protocol exists. | Unclear - unknown if baseline imbalances between groups | High risk |
| Macedo (2014) | Low risk – “A randomization approach using computer-generated random numbers…” | Unclear – no information provided | Unclear – no information provided | Low risk – no drop-outs reported | Unclear – no obvious selective reporting; it is difficult to judge however whether selective reporting is a problem, as no protocol exists. | Low risk - no other sources | Unclear |
| Miranda (2014) | Low risk – “computer-generated table to randomly allocate” | Low risk – “Identical plastic bottles containing the antibiotics or placebos were sent to one of the study coordinators (P.M.D.), who marked the code number of each subject on each bottle, according to the therapy assigned. Allocation concealment was assured by means of sequentially numbered drug containers of identical appearance. Study personnel, including the examiner (T.S.M.), the two operators, the investigator responsible for the data analysis (M.Fa.) and the participants were blinded to treatment assignment. Code breaking was performed after final statistical analysis. | Low risk – “Code breaking was performed after final statistical analysis” and placebos used. | Low risk – low drop-out rate (3.4%), while within-person design ensures balance across randomization groups. | Low risk - all outcomes reported | Low risk - no other sources | Low risk |
| Tsalikis (2014) | Low risk – “Randomization was generated  using randomization software” | High risk - randomization list kept by author | Low risk – “Neither therapists nor the examiner were aware of the treatment group” and placebos used. | Low risk – low drop-out rate (5.7%), while within-person design ensures balance across randomization groups. | Unclear – no obvious selective reporting; it is difficult to judge however whether selective reporting is a problem, as no protocol exists. | Low risk - no other sources | High risk |
| Wu (2015) | Unclear – “Participants were randomly divided into…” | Unclear – no information provided | High risk - not possible | Unclear - Eight of the 54 participants withdrew and no sample size calculation | Unclear – no obvious selective reporting; it is difficult to judge however whether selective reporting is a problem, as no protocol exists. | Low risk - no other sources | High risk |
| Koçak (2016) | Unclear – “Sixty patients with CP were randomly assigned into two groups…” | Low risk – “were presented to the senior author (SSH), who subsequently and “blindly” assigned each of the two ID cards to one and another unlabeled” | High risk –the first author (EK) knows the treatment mode and EK evaluates the clinical parameters | Low risk – no drop-outs reported | Unclear – no obvious selective reporting; it is difficult to judge however whether selective reporting is a problem, as no protocol exists. | Low risk - no other sources | High risk |
| Kumari (2016) | Low risk – “…using a computer-generated random table” | Unclear – no information provided | Low risk – “both investigators and individuals were masked to the groups allotted…All pre- and post-treatment clinical parameters were recorded by an examiner (ARP) who was masked to type of treatment received by patients” | Unclear – fifteen of 75 participants withdrew and not report detail reasons | Unclear – no obvious selective reporting; it is difficult to judge however whether selective reporting is a problem, as no protocol exists. | Low risk - no other sources | Unclear |
| Ramos (2016) | Low risk – “Randomization procedure was  performed by a single examiner, using a software program (SPSS Inc., Chicago, IL USA) by a computer-generated randomly permuted by (D.M.R) | Low risk – “Allocation was performed by A.B.N and was concealed with opaque envelopes” | Unclear – “The patients were assigned in two groups: SRP + aPDT (applications on 0, 3, 7, and 14 days posttherapy with a placebo capsule) and SRP + Doxy (systemic doxycycline 100 mg/day for 14 days with a first dose of 200 mg and the same protocol used on SRP + aPDT group, without light exposure), but we cannot judge if outcome assessor/ intervention-providers are blinded. | Low risk – no drop-outs reported | Unclear – no obvious selective reporting; it is difficult to judge however whether selective reporting is a problem, as no protocol exists. | Low risk - no other sources | Unclear |
