# Supplementary figures and images for: Effect of non-surgical periodontal therapy on glycemic control of type 2 diabetes mellitus: a systematic review and Bayesian network meta-analysis
Source: BMC Oral Health. 2019 Aug 6;19:176. doi: 10.1186/s12903-019-0829-y (PMC6685286; doi:10.1186/s12903-019-0829-y)

**Additional file 4.** Sensitivity analysis for informative uniform distribution.

**
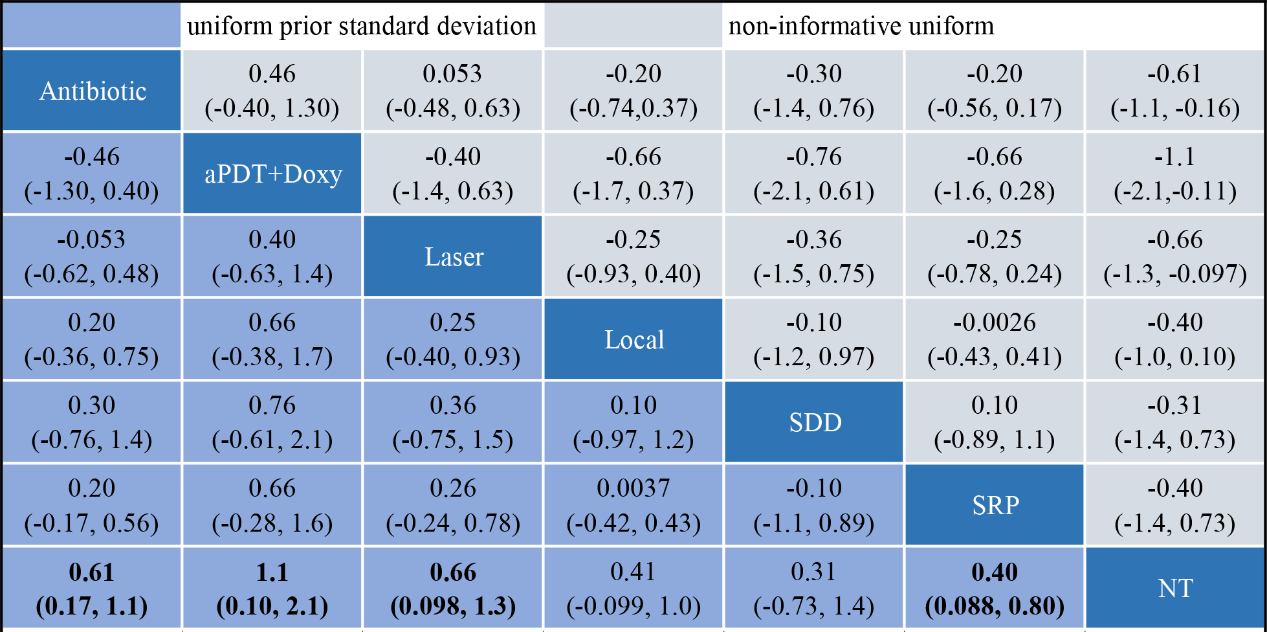
**

Supplement: Supplementary file 4 — Risk of bias summary: review authors' judgements about each risk of bias item for each included study. (DOCX 342 kb) [file 12903_2019_829_MOESM4_ESM.docx]

Additional file 5. Forest plot of changes in FPG


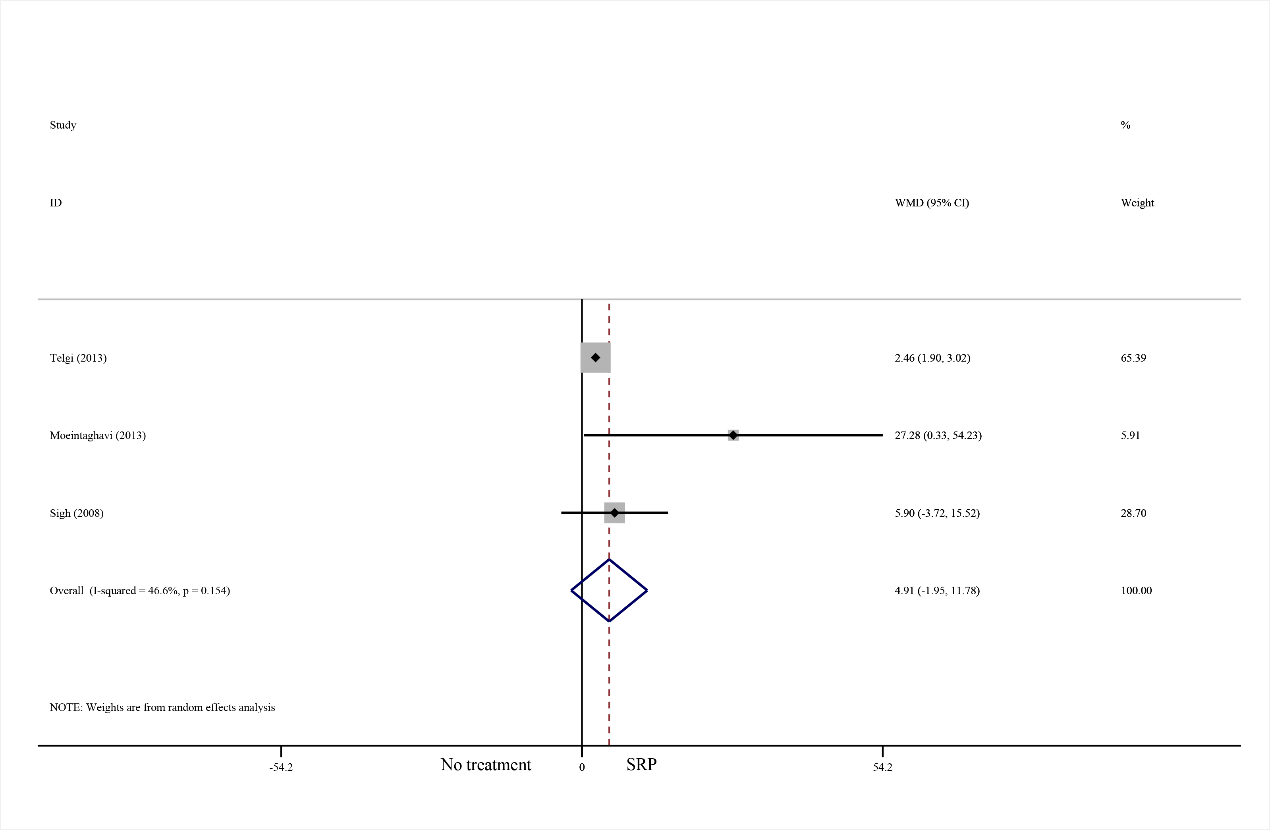


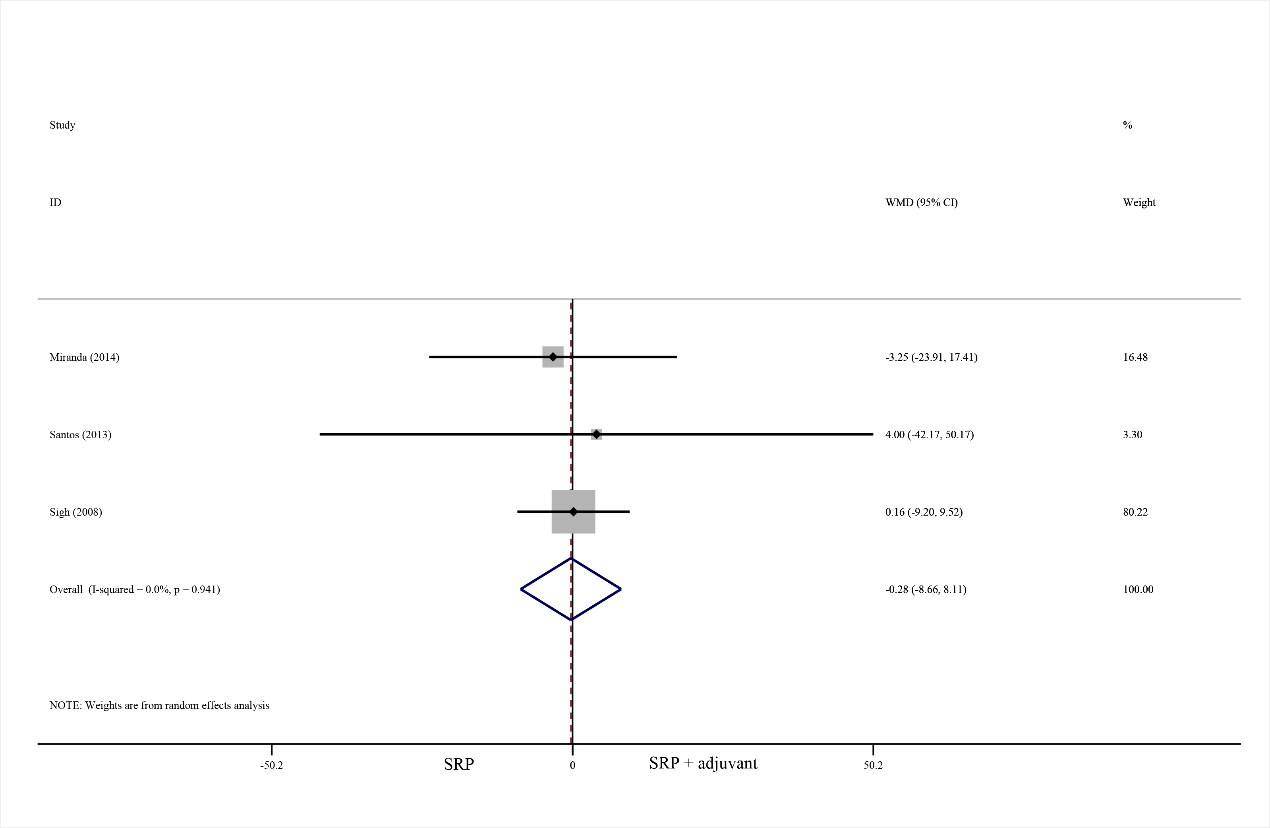

Supplement: Supplementary file 5 — Forest plot of changes in FPG. (DOCX 172 kb) [file 12903_2019_829_MOESM5_ESM.docx]
