# Supplementary material for: Effect of non-surgical periodontal therapy on glycemic control of type 2 diabetes mellitus: a systematic review and Bayesian network meta-analysis
Source: BMC Oral Health. 2019 Aug 6;19:176. doi: 10.1186/s12903-019-0829-y (PMC6685286; doi:10.1186/s12903-019-0829-y)
Supplement: Supplementary file 6 — Evaluation of model fit. (DOCX 14 kb) [file 12903_2019_829_MOESM6_ESM.docx]

Additional file 6. Evaluation of model ft in the included studies

| **Outcome** | **Dbar** | **Number of data points** |
| --- | --- | --- |
| HbA1c% | 26.75 | 29 |
