# Supplementary material for: Effect of non-surgical periodontal therapy on glycemic control of type 2 diabetes mellitus: a systematic review and Bayesian network meta-analysis
Source: BMC Oral Health. 2019 Aug 6;19:176. doi: 10.1186/s12903-019-0829-y (PMC6685286; doi:10.1186/s12903-019-0829-y)
Supplement: Supplementary file 7 — Sensitivity analysis. (DOCX 16 kb) [file 12903_2019_829_MOESM7_ESM.docx]

Additional file 7. The results of sensitivity analysis

| Treatment | Overall analysis | Sensitivity analysis (drop HbA1c% > 9) | Sensitivity analysis (drop HbA1c% < 7) |
| --- | --- | --- | --- |
| **SRP** |  |  |  |
| SRP + Antibiotic | 0.20 (-0.17, 0.56) | 0.23 (-0.19, 0.64) | 0.26 (-0.37, 0.85) |
| SRP + aPDT + Doxy | 0.66 (-0.28, 1.6) | 0.69 (-0.32, 1.7) | 0.72 (-0.56, 1.9) |
| SRP + Laser | 0.25 (-0.24, 0.78) | 0.19 (-0.50, 0.88) | 0.49 (-0.78, 1.7) |
| SRP + Local-delivery drugs | 0.0026 (-0.41, 0.43) | -0.000012 (-0.49, 0.49) | 0.10 (-2.0, 2.2) |
| No treatment | -0.40 (-0.80, -0.086) | -0.41 (-0.84, -0.061) | -0.43 (-0.98, 0.028) |
| SRP + SDD | -0.10 (-1.1, 0.89) | -0.10 (-1.1, 0.94) |  |
| **SRP + SDD** |  |  |  |
| SRP + Antibiotic | 0.30 (-0.76, 1.4) | 0.34 (-0.79, 1.4) |  |
| SRP + aPDT + Doxy | 0.76 (-0.61, 2.1) | 0.79 (-0.65, 2.2) |  |
| SRP + Laser | 0.36 (-0.75, 1.5) | 0.29 (-0.95, 1.5) |  |
| SRP + Local-delivery drugs | 0.10 (-0.97, 1.2) | 0.10 (-1.0, 1.2) |  |
| No treatment | -0.31 (-1.4, 0.73) | -0.31 (-1.4, 0.77) |  |
| **No treatment** |  |  |  |
| SRP + Antibiotic | 0.61 (0.16, 1.11) | 0.64 (0.15, 1.2) | 0.69 (-0.0059, 1.4) |
| SRP + aPDT + Doxy | 1.1 (0.11, 2.1) | 1.1 (0.072, 2.2) | 1.1 (-0.15, 2.5) |
| SRP + Laser | 0.66 (0.097, 1.3) | 0.59 (-0.15, 1.4) | 0.92 (-0.37, 2.2) |
| SRP + Local-delivery drugs | 0.40 (-0.10, 1.0) | 0.40 (-0.17, 1.1) | 0.53 (-1.6, 2.7) |
| **SRP + Local-delivery drugs** |  |  |  |
| SRP + Antibiotic | 0.20 (-0.37, 0.74) | 0.23 (-0.42, 0.86) | 0.16 (-2.1, 2.4) |
| SRP + aPDT + Doxy | 0.66 (-0.37, 1.7) | 0.69 (-0.44, 1.8) | 0.62 (-1.9, 3.1) |
| SRP + Laser | 0.25 (-0.40, 0.93) | 0.19 (-0.65, 1.0) | 0.39 (-2.1, 2.8) |
| **SRP + Laser** |  |  |  |
| SRP + Antibiotic | -0.053 (-0.63, 0.48) | 0.047 (-0.77, 0.83) | -0.23 (-1.3, 0.86) |
| SRP + aPDT + Doxy | 0.40 (-0.63, 1.4) | 0.50 (-0.71, 1.7) | 0.23 (-1.3, 1.8) |
| **SRP + aPDT + Doxy** |  |  |  |
| SRP + Antibiotic | -0.46 (-1.3, 0.40) | -0.46 (-1.4, 0.45) | -0.46 (-1.60, 0.64) |
