# Supplementary material for: Effect of non-surgical periodontal therapy on glycemic control of type 2 diabetes mellitus: a systematic review and Bayesian network meta-analysis
Source: BMC Oral Health. 2019 Aug 6;19:176. doi: 10.1186/s12903-019-0829-y (PMC6685286; doi:10.1186/s12903-019-0829-y)
Supplement: Supplementary file 8 — Data extraction table of this network meta-analysis. (DOCX 16 kb) [file 12903_2019_829_MOESM8_ESM.docx]

Additional file 8. The data of HbA1c%

| Study | x1 | s1 | n1 | x2 | s2 | n2 | treatment | t1 | t2 |
| --- | --- | --- | --- | --- | --- | --- | --- | --- | --- |
| Sigh | 0.8 | 0.66 | 15 | 0.6 | 0.66 | 15 | antibiotic vs. SRP | antibiotic | SRP |
| Sigh | 0.8 | 0.66 | 15 | -0.02 | 0.72 | 15 | antibiotic vs. NT | antibiotic | NT |
| Gilowski | 0 | 0.9 | 17 | 0.1 | 1.41 | 17 | SDD vs. SRP | SDD | SRP |
| Moeintaghavi | 0.74 | 1.18 | 22 | -0.25 | 2.05 | 18 | SRP vs. NT | SRP | NT |
| Gaikwad | 1.38 | 0.83 | 25 | 0.95 | 1.05 | 25 | antibiotic vs. SRP | antibiotic | SRP |
| Pradeep | 0.02 | 0.12 | 17 | 0.03 | 0.13 | 18 | local vs. SRP | local | SRP |
| Santos | 0.78 | 3.5 | 19 | 0.68 | 2.5 | 18 | local vs. SRP | local | SRP |
| Telgi | 0.58 | 0.27 | 20 | 0.004 | 0.12 | 20 | SRP vs. NT | SRP | NT |
| Macedo | 0.87 | 0.9 | 15 | 0.41 | 0.84 | 15 | aPDT + Doxy vs. antibiotic | aPDT + Doxy | antibiotic |
| Miranda | -0.07 | 1.83 | 29 | 0.05 | 1.67 | 27 | antibiotic vs. SRP | antibiotic | SRP |
| Tsalikis | 0.08 | 0.58 | 31 | -0.07 | 0.88 | 35 | antibiotic vs. SRP | antibiotic | SRP |
| Wu | 0.01 | 0.19 | 23 | 0.01 | 0.16 | 23 | SRP vs. NT | SRP | NT |
| Kocak | 0.41 | 0.19 | 30 | 0.22 | 0.25 | 30 | laser vs. SRP | laser | SRP |
| Kumari | 0.04 | 0.14 | 30 | 0.03 | 0.13 | 30 | local vs. SRP | local | SRP |
| Ramos | 0.99 | 1 | 15 | 0.76 | 0.73 | 15 | aPDT vs. antibiotic | laser | antibiotic |

The data of FPG

| Study | x1 | s1 | n1 | x2 | s2 | n2 | treatment | t1 | t2 |
| --- | --- | --- | --- | --- | --- | --- | --- | --- | --- |
| Sigh | 4 | 14.45 | 15 | -1.9 | 12.35 | 15 | SRP vs NT | SRP | NT |
| Sigh | 4.16 | 11.55 | 15 | 4 | 14.45 | 15 | SRP+adjuvant vs SRP | SRP+adjuvant | SRP |
| Moeintaghavi | 17.5 | 48.91 | 22 | -9.78 | 38.02 | 18 | SRP vs NT | SRP | NT |
| Telgi | 2.88 | 1.07 | 20 | 0.42 | 0.71 | 20 | SRP vs NT | SRP | NT |
| Santos | 5.4 | 65.09 | 19 | 1.4 | 77.31 | 18 | SRP+adjuvant vs SRP | SRP+adjuvant | SRP |
| Miranda | 5.46 | 38.3 | 29 | 8.71 | 40.41 | 27 | SRP+adjuvant vs SRP | SRP+adjuvant | SRP |
